# Helium Plasma Radiofrequency for Aesthetic Subdermal Treatment: A Systematic Review and Meta-Analysis

**DOI:** 10.1093/asjof/ojag092

**Published:** 2026-05-22

**Authors:** Sachin M Shridharani, Aris Sterodimas, Manya R Harsch, Kari A Larson

## Abstract

Helium plasma radiofrequency (RF) technology (Renuvion, Apyx Medical, Clearwater, FL) has emerged as a minimally invasive option for treating skin laxity, either as a standalone procedure or as an adjunct to liposuction. While clinical adoption has expanded rapidly, the literature remains heterogeneous, with variable study designs and outcomes. To comprehensively evaluate the safety and performance of helium plasma RF for aesthetic subdermal applications through systematic review and meta-analysis. A systematic search was performed from initial device clearance through May 2025, following PRISMA guidelines. Eligible studies included randomized controlled trials, prospective cohorts, retrospective reviews, and case series with ≥10 patients undergoing aesthetic subdermal treatment with helium plasma RF. Safety outcomes were tabulated and weighted incidence rates calculated. Random-effects meta-analyses were performed to generate pooled estimates for performance endpoints, including patient satisfaction, investigator global aesthetic improvement scale (GAIS), patient GAIS, and independent photographic review scores. Thirty-four studies across 33 publications encompassing 3508 patients were included. Pooled complication rates were 5% for helium plasma RF-only, 8% for helium plasma RF + liposuction, and 15% for combination procedures involving multimodal excisional surgery; differences between groups were not statistically significant (*P* = .14). The pooled patient satisfaction rate was 92%. Meta-regression demonstrated higher satisfaction with longer follow-up durations (*P* = .003). This systematic review and meta-analysis suggest that helium plasma RF is associated with a favorable safety profile and high rates of patient satisfaction and aesthetic improvement in subdermal applications.

**Level of Evidence**: 4 (Therapeutic) 
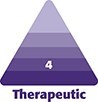

Skin laxity is a common aesthetic concern resulting from intrinsic aging, photoaging, weight fluctuations, pregnancy, and other physiological changes that affect the dermis and subcutaneous soft tissues.^1^ These changes lead to decreased collagen and elastin production, fragmentation of elastic fibers, and remodeling of the extracellular matrix, collectively reducing the structural integrity of the skin.^2–4^ Clinically, this manifests as visible laxity, sagging, and contour irregularities that can impact both appearance and patient’s self-confidence.^1^

Historically, excisional surgical procedures such as abdominoplasty, brachioplasty, and lower body lifts were considered the standard of care for treating excess skin. While effective, these surgeries are highly invasive and associated with visible scarring, prolonged recovery times, and a high risk of complications such as wound dehiscence and hematoma.^5^ As patient demand has shifted toward less invasive options with shorter downtime, minimally invasive technologies have gained prominence in the aesthetic field.^6–8^

Energy-based devices are now well established for subdermal and subcutaneous tissue coagulation and contraction of the fibroseptal network (FSN), a connective tissue lattice that anchors the skin to underlying fascia.^7,9^ These devices deliver thermal energy, inducing collagen denaturation and contraction while also triggering a delayed healing response that stimulates neocollagenesis, elastin regeneration, and dermal remodeling over time.^10,11^ Used either as standalone procedures or as an adjunct to liposuction, energy-based treatments provide a less invasive alternative to excisional surgery for patients with mild to moderate laxity.^12,13^

The Renuvion system (Apyx Medical, Clearwater, FL), also historically referred to as J-Plasma, represents a novel advancement in minimally invasive energy-based devices. This helium plasma radiofrequency (RF) technology combines RF energy with helium plasma to deliver focused energy and controlled tissue effects.^14^

The device generates a high-energy plasma stream by passing helium gas over an energized electrode within a single-use handpiece. The plasma stream serves 2 essential functions:

1. Efficient energy delivery: helium ionizes at a low-voltage threshold, enabling the RF current to be precisely focused within target tissues.
2. Rapid tissue cooling: simultaneous energy delivery and cooling minimize thermal spread, allowing coagulation and contraction without bulk heating of superficial layers as seen with other devices.

This dual mechanism enables instantaneous FSN contraction with progressive tissue remodeling, resulting in both immediate contraction and gradual improvements over time. During treatment, the handpiece is introduced percutaneously into the subcutaneous plane, delivering energy to the FSN. Collagen denatures at approximately 66.8°C; helium plasma RF raises local tissue temperatures to 80 to 90°C, resulting in rapid denaturation and contraction of collagen fibers.^14^ Upon thermal denaturation, collagen contracts to roughly one-third of its original length as its triple-helical structure unravels into a coiled form.^15,16^ This contraction draws the overlying skin closer to underlying structures, visibly improving laxity and contour.

Since its initial FDA surgical clearance in 2009, helium plasma RF has been adopted across a range of aesthetic applications, including standalone subdermal tissue contraction and as an adjunct to liposuction. It is used in anatomical areas all across the body.

Clinical studies have reported improvements in skin laxity, contour, and patient satisfaction. However, the available literature remains heterogeneous, with variability in study design, methodology, outcome measures, and terminology. Most publications to date are case series or retrospective analyses, with fewer randomized controlled trials or comparative studies available. While these studies provide important evidence supporting the safety and performance of helium plasma RF, the variability in study design, methodology, and outcome measures makes it difficult to draw comprehensive conclusions regarding safety, performance, and optimal patient selection based on any single study alone.

Given its rapid clinical adoption, a systematic and quantitative assessment of helium plasma RF is warranted. Systematic reviews provide a structured approach to identify, appraise, and synthesize available evidence, while meta-analyses allow for the pooling of data across studies to generate more precise estimates of treatment effects and complication rates. Together, these methods can provide a robust evaluation of both the breadth and strength of the current clinical literature.

This study was designed to systematically review and, where appropriate, quantitatively synthesize published clinical data on the use of helium plasma RF for aesthetic subdermal applications. By consolidating the current evidence through both systematic review and meta-analysis, this study provides clinicians and patients with a comprehensive, evidence-based understanding of helium plasma RF's role within the modern aesthetic treatment landscape.

## METHODS

### Overview

This systematic review and meta-analysis were conducted in accordance with the Preferred Reporting Items for Systematic Reviews and Meta-Analyses (PRISMA) guidelines.^17,18^ A protocol was developed a priori, with input from physician experts (S.M.S. and A.S.), to define the literature search, study selection, data extraction, bias assessment, and statistical analyses. This protocol was not registered in a public registry, as the review was conducted as part of an evidence synthesis initiative; however, all eligibility criteria and analysis methods were prespecified prior to study screening and extraction.

The objective was to identify, appraise, and synthesize all published clinical data evaluating the use of helium plasma RF for aesthetic subdermal applications. Safety outcomes were tabulated across studies, with ranges of adverse events (AEs) reported and weighted incidence rates calculated to provide an overall summary of safety findings. In parallel, a meta-analysis was performed to generate pooled estimates of key performance outcomes, including patient satisfaction, Investigator Global Aesthetic Improvement Scale (GAIS), Patient GAIS, and independent photographic review (IPR) scores. Other safety and effectiveness outcomes were also tabulated.

### Inclusion/Exclusion Criteria

Eligible studies included randomized controlled trials, prospective cohort studies, retrospective reviews, and case series with a minimum of 10 patients. Studies were required to involve human subjects undergoing aesthetic subdermal treatment with helium plasma RF, either as a standalone procedure or as an adjunct to liposuction. Only English-language articles and conference abstracts providing sufficient clinical detail for analysis were included.

Exclusion criteria were preclinical studies, case reports with fewer than 10 patients, reviews, editorials, expert opinion pieces, and studies focused on dermal resurfacing or nonsubdermal surgical applications (eg, gynecological malignancies).

### Data Sources and Search Strategy

A comprehensive literature search was conducted on May 21, 2025 across PubMed, Europe PMC, ScienceDirect, and Google Scholar. The search encompassed all publications from initial regulatory clearance through the search date. Boolean operators and synonyms were used to capture all relevant studies, including combinations of terms such as “Renuvion,” “J-Plasma,” “helium plasma,” “radiofrequency,” “skin laxity,” “liposuction,” “body contouring,” and “aesthetic surgery.”

**Figure 1.**
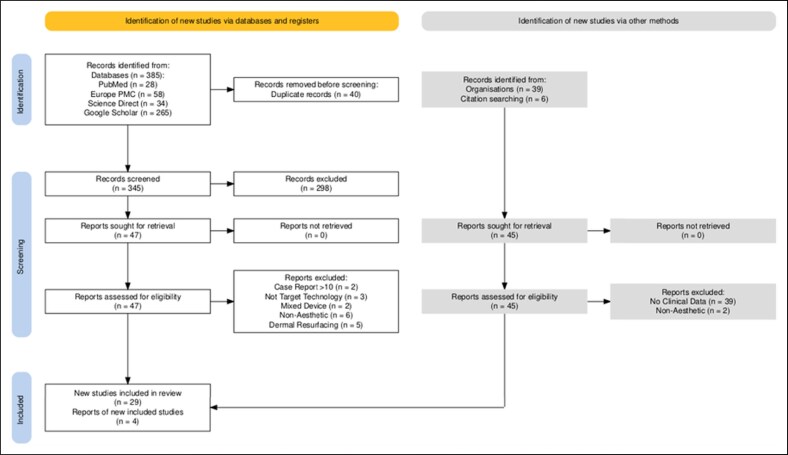
PRISMA 2020 flow diagram summarizing identification, screening, eligibility assessment, and inclusion of studies in the systematic review and meta-analysis. The diagram was generated using the PRISMA2020 R package and Shiny app.^19^

Reference lists of included articles and related reviews were also screened manually to identify additional sources. Search results were imported into DistillerSR (Ottawa, Canada) for de-duplication and screening. Titles/abstracts and full-text screening were performed by a single reviewer (K.A.L.) using prespecified eligibility criteria; therefore, no inter-reviewer disagreement resolution process was applicable. Eligibility was determined based on prespecified inclusion and exclusion criteria, including study type, sample size, treatment indication, and availability of safety or performance outcomes relevant to aesthetic subdermal helium plasma RF use. Reasons for exclusion at the full-text stage were documented within DistillerSR. A list of excluded full-text studies and reasons for exclusion is provided in the Appendix, available online at https://doi.org/10.1093/asjof/ojag092. The PRISMA flow diagram (Figure 1) illustrates the number of records identified, screened, excluded, and included in the final qualitative and quantitative analyses. Included evidence is reported as 33 publications representing 34 distinct studies; 1 publication contributed 2 separate study cohorts.

### Data Extraction and Quality Assessment

Data were extracted from eligible full-text studies by a single reviewer (K.A.L.) using a standardized form and included study design, sample size, anatomical treatment areas, patient demographics, intervention details (including treatment parameters), and follow-up duration. Reported outcomes included safety events and performance measures, such as AEs, GAIS, IPR, patient satisfaction rates, and other safety or performance outcomes specific to individual studies that were not suitable for pooled analysis. All extracted data were verified against the source publication prior to inclusion in quantitative synthesis. When required data were unclear or not explicitly stated, values were recorded as not reported, and the study was retained for qualitative synthesis when appropriate.

Study quality and risk of bias were evaluated using validated tools: the Cochrane Risk of Bias 2 tool^20^ for randomized controlled trials and the ROBINS-I tool^21^ for nonrandomized studies. Each domain was rated as low, moderate, serious, or critical risk of bias with written justifications documented for transparency. Risk of bias assessments were performed by the same reviewer. Further information is provided in the Appendix via Supplemental Tables 1, 2. Certainty of evidence across outcomes was not formally assessed using a framework such as GRADE; however, study-level methodological quality was evaluated using RoB 2 and ROBINS-I tools, and heterogeneity and publication bias were assessed through *I*^2^ statistics and funnel plot/Egger's testing.

### Meta-Analysis

The meta-analysis was conducted by an independent statistician using a random-effects model to account for heterogeneity between studies. For each endpoint, proportions and 95% CIs were calculated for individual studies using exact binomial methods, with pooled estimates generated through the random-effects model. Forest plots display study-level results and pooled proportions, while Egger's test with funnel plots using Freeman–Tukey transformed proportions was used to detect potential publication bias. Funnel plot asymmetry was assessed visually, and Egger's regression test was applied when ≥10 studies were available for a given endpoint.

Sensitivity analyses included a leave-one-out approach, sequentially excluding each study to assess its influence on the overall pooled result. Subgroup analyses were performed by geographic region (United States vs other countries) and funding source (industry-sponsored vs investigator-initiated or independent studies). Meta-regression was performed to assess the impact of average follow-up duration, and if significant, a cumulative forest plot was generated to visualize its effect.

Adverse event data were analyzed to characterize clinically meaningful safety outcomes. Expected treatment effects (ETEs), such as transient edema, ecchymosis, bruising, temporary nerve effects, and systemic events related to anesthesia (eg, nausea), were excluded from pooled analysis to focus on clinically significant or nonexpected events. When studies reported an overall complication rate, that value was used directly. If an overall rate was not provided, individual AE categories reported within the study were summed to estimate the overall complication rate. This conservative approach may overestimate the proportion of subjects with complications, as some individuals may have experienced multiple events counted separately.

Under the guidance of physician experts (S.M.S. and A.S.), AEs were categorized into predefined domains to allow for meaningful analysis and aggregation across studies. Weighted incidence rates were calculated for each domain where sufficient data were reported to provide a summary of the safety profile. Adverse event analysis was conducted across 3 procedural categories: helium plasma RF-only subdermal applications, helium plasma RF + liposuction, and combination procedures (eg, helium plasma RF used with multimodal excisional surgery).

The meta-analysis of overall complication rates included studies that either reported a total complication rate or provided sufficient detail to calculate one by summing individual AE categories. This conservative approach may overestimate the true proportion of patients experiencing a complication, as a single patient could have multiple events counted separately. Because the pooled complication rate represents a modeled estimate derived from study-level weighting, it will not equal the arithmetic mean of individual study rates. The random-effects model accounts for between-study variability and sample size, whereas the simple average does not, leading to apparent differences between these values. Accordingly, the sum of weighted average complication rates within each subgroup will not equal the pooled complication rate, which represents a modeled proportion derived from random-effects weighting rather than arithmetic aggregation.

Each study was assigned a level of evidence according to the National Health and Medical Research Council (NHMRC) classification system. Safety outcomes were summarized descriptively, while pooled proportions with 95% CIs were reported for performance outcomes. Heterogeneity was quantified using the *I*^2^ statistic, with thresholds of 25%, 50%, and 75% indicating low, moderate, and high heterogeneity, respectively. Statistical significance was defined as *P* < .05 for all analyses.

## RESULTS

### Study Selection and Characteristics

The systematic search identified 385 records, of which 92 full-text articles were reviewed and 33 publications met inclusion criteria, representing 34 distinct studies and encompassing 3508 treated subjects. One publication reported outcomes from 2 separate study cohorts and was therefore counted as 2 studies. The PRISMA flow diagram (Figure 1) summarizes the screening and selection process.

Included studies encompassed a mix of randomized controlled trials (Level II, *n* = 2),^22,23^ comparative nonrandomized studies (Level III-2, *n* = 7),^24-30^ and case series or single-surgeon cohorts without control groups (Level IV, *n* = 25), with both independent or investigator-initiated research (*n* = 18) and industry-sponsored studies (*n* = 16) represented. Tables 1 and 2 summarize the study characteristics, NHMRC levels of evidence, and safety and effectiveness outcomes. Expanded study findings and characteristics including treatment parameters are provided in the Appendix via Supplemental Tables 3, 4.

**Table 1.** Summary of Included Clinical Studies (Arshad–Ouf) Evaluating Helium Plasma Radiofrequency for Aesthetic Subdermal Applications

Study	Country	Design/level	N	Application/comparator	Safety	Efficacy
Arshad et al (2023)^31^	USA	Case series (IV)	20	BBL + HPRF	No device AEs	↑ contour; high satisfaction
Barone et al (2025)^22^	Italy	RCT (II)	76	Abdominoplasty ± HPRF vs abdominoplasty	No ↑ vs control	↑ BODY-Q; improved aesthetic ratings
De La Cruz (2024)^24^	USA	Cohort (III-2)	88	UAL vs SEAL + HPRF	Low AEs	Favorable outcomes (qualitative)
De Souza and De Souza (2022)^32^	USA	Case series (IV)	49	BBL + HPRF	No SAEs	↑ tightness; high satisfaction
Doolabh (2019)^33^	USA	Case series (IV)	32	Subdermal HPRF post-lipo	No device AEs	↑ contraction; skin redraping
Doolabh and Ruff (2020)^34^	USA	Case series (IV)	15	Neck/submental HPRF	2 AEs; no SAEs	↓ cervicomental angle; ↑ IPR
Driscoll et al (2024)^35^	USA	Case series (IV)	180	Multi-area HPRF ± VASER	13% AEs; no reop	↑ skin redraping
Hoyos (2025^36^)	Columbia	Case series (IV)	174	HD lipo + HPRF	3.5% AEs; no reop	High satisfaction
Ibrahiem (2022)^25^	Egypt	Cohort (III-2)	176	VASER ± RFAL vs VASER + HPRF vs VASER	No diff vs groups	Renuvion ↑ outcomes vs VASER
Khedr and Elshawadfy (2024)^37^	Egypt	Case series (IV)	46	Lipo + HPRF	Minor AEs	82.6% satisfied; ↑ photos
Kluska et al (2024)^26^	USA	Cohort (III-2)	465	HPRF vs bipolar RF	↓ AEs vs bipolar RF	↑ satisfaction; ↓ time; ↑ area reduction
Lacerna (2025)^38^	USA	Case series (IV)	26	Brow lift	11.5% AEs; no major	↑ IPR; ↑ satisfaction
Mowlavi et al (2020)^39^	USA	Case series (IV)	14	UAL + HPRF	No AEs	100% satisfaction
Nunez Villar (2024^40^)	Peru	Case series (IV)	220	Lipotransfer	Mild AEs (5%)	Maintained contour
Ouf et al (2024)^23^	Egypt	RCT (II)	45	Lipo vs VASER vs + HPRF	Minor AEs only	↑ redundancy, ↑ Cutometer, ↑ satisfaction

Detailed study-level safety and efficacy findings are provided in Supplemental Table 3.

HPRF, helium plasma radiofrequency; AE, adverse event; SAE, serious adverse event; UAL, ultrasound-assisted liposuction; RF, radiofrequency; GAIS, Global Aesthetic Improvement Scale; IPR, Independent Photographic Review; HD, high-definition; SEAL, synchronous energy-assisted liposuction. ↑ indicates improvement. ↓ indicates reduction. “Not reported” indicates efficacy outcomes were not formally assessed.

**Table 2.** Summary of Included Clinical Studies (Ruff–Zorrilla) Evaluating Helium Plasma Radiofrequency for Aesthetic Subdermal Applications

Study	Country	Design/level	N	Application/comparator	Safety	Efficacy
Ruff et al (2023)^12^	USA	Prospective (IV)	65	Neck/submental	No SAEs	↑ IPR, GAIS, lift, satisfaction
Ruff et al (2020)^41^	USA	Case series (IV)	185	Multi-area HPRF	Mild AEs; rare SAEs	↑ contour; high satisfaction
Ruff et al (2024)^27^	USA	Cohort (III-2)	150	UAL vs + HPRF	No SAEs	↑ perceived contraction
Ruff et al (2022)^28,42^ (Gyn)	USA	Case series (IV)	84	Lipo + HPRF	Few AEs	Not reported
Ruff et al (2022)^28^ (AE)	USA	Cohort (III-2)	192	HPRF ± UAL vs HPRF	Seromas (combo only)	Not reported
Shridharani et al (2024)^43^	USA	Case series (IV)	483	Post-lipo HPRF	Low AEs; no major	Not reported
Shridharani (2022^44^)	USA	Case series (IV)	47	Subdermal HPRF	ETEs only	↑ subjective improvement
Skenderian et al (2023)^45^	USA	Case series (IV)	115	Hi-def abdomen	Not systematically reported	Anecdotal benefit
Sterodimas et al (2025)^46^ (breast)	Greece	Case series (IV)	15	Breast	No AEs	↑ IPR, GAIS, Breast-Q
Sterodimas et al (2025)^47^ (lower eyelid)	Greece	Case series (IV)	16	Eyelid	1 mild AE	↑ laxity, GAIS, satisfaction
Sterodimas et al (2025)^48^ (forehead)	Greece	Case series (IV)	30	Forehead	No SAEs	100% satisfaction
Tambasco et al (2024)^49^ (chest)	Italy	Case series (IV)	300	Gynecomastia	AEs ↑ with severity	100% satisfaction
Tambasco et al (2025)^50^ (639 pts)	Italy	Case series (IV)	639	UAL + HPRF	Low AEs	91% ↑ contour
Tambasco et al (2025)^51^ (thigh)	Italy	Case series (IV)	21	Thighplasty + HPRF	Minor AEs	Qualitative improvement
Tambasco et al (2024)^49,52^ (Lipoabd)	Italy	Case series (IV)	100	Lipoabdominoplasty	Minor AEs	High satisfaction
Troell (2025^53^)	USA	Case series (IV)	58	VASER + HPRF (face/neck)	No SAEs	95.5% GAIS improvement
Vanek (2025)^29^	USA	Cohort (III-2)	77	UAL vs UAL ± HPRF	No diff vs control	Not reported
Zorrilla et al (2023)^30^	USA	Cohort (III-2)	302	Aesthetic procedures ± HPRF	Low AEs	Not reported

Detailed study-level safety and efficacy findings are provided in Supplemental Table 3.

HPRF, helium plasma radiofrequency; AE, adverse event; SAE, serious adverse event; UAL, ultrasound-assisted liposuction; RF, radiofrequency; GAIS, Global Aesthetic Improvement Scale; IPR, Independent Photographic Review; HD, high-definition. ↑ indicates improvement. “Not reported” indicates efficacy outcomes were not formally assessed.

**Figure 2.**
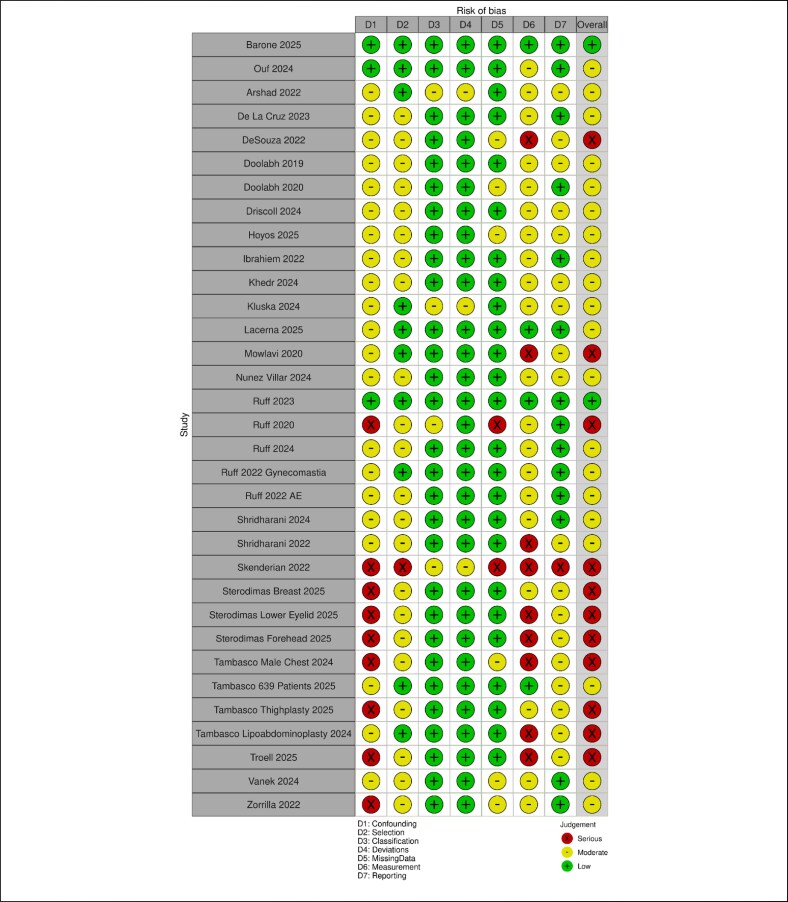
Risk of bias assessment across included studies. Study-level risk of bias across domains using RoB 2 and ROBINS-I tools. Colors indicate risk level (green = low, yellow = moderate/some concerns, red = serious/high).

**Figure 3.**
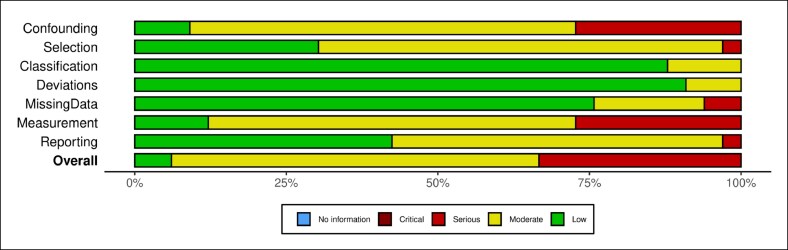
Summary risk of bias across included studies by domain. Proportion of studies rated as low (green), moderate (yellow), or serious (red) risk of bias across each domain using RoB 2 and ROBINS-I tools.

Risk of bias assessments demonstrated variability in methodological quality across included studies. Among randomized controlled trials, one study was assessed as low risk of bias, while the second demonstrated some concerns. Among nonrandomized studies, the majority were rated as moderate risk of bias, primarily due to retrospective design, lack of control groups, and reliance on subjective outcome measures. Notably, approximately one-third of included studies were assessed as having serious risk of bias, which is consistent with the predominance of study designs in this field. These ratings are largely driven by uncontrolled confounding, absence of comparator groups, and nonstandardized or unblinded outcome assessment. Risk of bias visualizations were generated using the robvis web application (Figures 2, 3). Detailed domain-level assessments are presented in the Appendix, Supplemental Tables 1 and 2. Despite the limitations inherent to observational designs, the cumulative body of evidence reflects real-world clinical experience across diverse geographic regions, practice settings, and patient populations.

### Safety Profile From Systematic Review

Safety findings from the systematic review are summarized in Tables 1 and 2. Expected treatment effects, such as transient edema, ecchymosis, bruising, and temporary nerve effects, were common and typically resolved spontaneously or with conservative management. For the helium plasma RF-only group, reported AEs were generally mild and transient. Weighted average rates included 6.3% dermatological reactions (eg, contact dermatitis), 5.9% transient neurological effects (eg, marginal mandibular nerve impact), 4.4% fluid accumulation events (seroma or hematoma), 3.8% wound healing complications or local tissue integrity (eg, epidermal lysis, abscess/infection, scarring), 3.6% skin integrity and fibrotic tissue complications (subcutaneous induration), and 1.5% gas-related/helium plasma-specific events (gas buildup) (Table 3).

**Table 3.** Systematic Review Complication Rates and Weighted Rates—Helium-Plasma RF-Only

			Percent of Patients with an Adverse Event													
First Author, Year	Country	Sample size	Cardiopulmonary and vascular complications	Contour irregularities	Dermatological reactions	Edema/lymphatic complications	Fluid accumulation events	Gas-related/helium plasma-specific events	Neurologic effects/nerve impact	Ophthalmologic/periorbital events	Pigment changes	Skin integrity and fibrotic tissue complications	Wound healing complications/local tissue integrity	Article reported no AEs observed	Article reported only ETEs	Overall AE complication
De Souza and De Souza (2022)^32^	USA	49	—	—	—	—	—	—	—	—	—	—	—	No AEs		0.0%
Doolabh (2019)^33^	USA	15	—	—	—	—	—	—	6.7%	—	—	—	6.7%	—	—	13.3%*
Lacerna (2025)^38^	USA	26	—	—	—	—	3.8%	—	11.5%	—	—	—	—	—	—	11.5%
Ruff et al (2023)^12^	USA	65	—	—	—	—	4.6%	1.5%	6.2%	—	—	4.6%	3.1%	—	—	20.0%*
Shridharani (2022^44^)	USA	47	—	—	—	—	—	—	2.1%	—	—	2.1%	—	—	—	4.3%*
Sterodimas et al (2025)^46^ (breast)	Greece	15	—	—	—	—	—	—	—	—	—	—	—	No AEs	—	0.0%
Sterodimas et al (2025)^48^ (forehead)	Greece	30	—	—	—	—	—	—	—	—	—	—	—	—	ETEs only	0.0%
Sterodimas et al (2025)^47^ (lower eyelid)	Greece	16	—	—	6.3%	—	—	—	—	—	—	—	—	—	—	6.3%*
No. of studies			0	0	1	0	2	1	4	0	0	2	2	—	—	8
Study average			0	0	6.3%	0	4.2%	1.5%	6.6%	0	0	3.3%	4.9%	—	—	6.9%
Weighted average			0	0	6.3% (1/16)	0	4.4% (4/91)	1.5% (1/65)	5.9% (9/153)	0	0	3.6% (4/112)	3.8% (3/80)	—	—	8.0% (21/263)

^*^Denotes imputed overall complication by adding up all AEs & ETEs. AE, adverse event; ETEs, expected treatment effect; RF, radiofrequency.

For the helium plasma RF + liposuction group, reported AEs were generally minor and resolved spontaneously or with conservative management. Weighted average rates included 5.9% fluid accumulation events (seroma or hematoma), 5.0% edema/lymphatic complication (postoperative lymphedema), 4.3% contour irregularities (eg, revision required, residual skin laxity, asymmetry), 3.3% gas-related/helium plasma-specific events (gas buildup), 1.5% pigmentary changes, 1.4% transient neurological effects (eg, marginal mandibular nerve impact), 1.1% wound healing complications or local tissue integrity (eg, necrosis, infection, open wound), 0.9% cardiopulmonary and vascular complications (anemia), 0.9% dermatological reactions (skin rash), and 0.8% skin integrity and fibrotic tissue complications (subcutaneous induration, burn, fibrosis, scarring) (Table 4).

**Table 4.** Systematic Review Complication Rates and Weighted Rates—Helium Plasma RF + Liposuction

			Percent of patients with an adverse event													
First author, year	Country	Sample size	Cardiopulmonary and vascular complications	Contour irregularities	Dermatological reactions	Edema/lymphatic complications	Fluid accumulation events	Gas-related/helium plasma-specific events	Neurologic effects/nerve impact	Ophthalmologic/periorbital events	Pigment changes	Skin integrity and fibrotic tissue complications	Wound healing complications/local tissue integrity	Article reported no AEs observed	Article reported only ETEs	Overall AE complication
De La Cruz (2024)^24^	USA	25	—	—	—	—	8.0%	4.0%	—	—	—	—	—	—	—	12.0%*
Doolabh (2019)^33^	USA	32	—	—	—	—	—	—	—	—	—	—	—	No AEs	—	0.0%
Driscoll et al (2024)^35^	USA	180	—	3.3%	—	5.0%	3.9%	—	0.6%	—	—	0.6%	—	—	—	13.3%
Hoyos (2025^36^)	Columbia	96	—	2.1%	—	—	—	3.1%	—	—	—	—	—	—	—	5.2%*
Ibrahiem (2022)^25^	Egypt	66	—	7.6%	—	—	1.5%	—	—	—	—	4.5%	—	—	—	13.6%*
Khedr and Elshawadfy (2024)^37^	Egypt	46	—	2.2%	—	—	6.5%	—	—	—	—	—	—	—	—	8.7%*
Kluska et al (2024)^26^	USA	229	0.9%	—	0.9%	—	4.8%	—	—	—	—	0.9%	1.3%	—	—	8.7%*
Mowlavi et al (2020)^39^	USA	14	—	—	—	—	—	—	—	—	—	—	—	No AEs	—	0.0%
Nunez (2024^40^)	Peru	220	—	0.5%	—	—	—	—	—	—	—	—	—	—	—	0.5%*
Ouf et al (2024)^23^	Egypt	15	—	6.7%	—	—	20.0%	—	—	—	13.3%	6.7%	6.7%	—	—	53.3%*
Ruff et al (2020)^41^ CR1	USA	37	—	—	—	—	—	—	—	—	—	—	—	—	ETEs only	0.0%
Ruff et al (2020)^41^ CR2	USA	148	—	—	—	—	10.8%	—	—	—	—	—	—	—	—	10.8%*
Shridharani et al (2024)^43^	USA	483	—	—	—	—	4.6%	—	0.4%	—	—	0.2%	1.4%	—	—	6.6%
Tambasco et al (2025)^50^ (639 pts)	Italy	639	—	—	—	—	6.7%	—	—	—	1.3%	—	0.6%	—	—	8.6%*
Troell (2025^53^)	USA	58	—	22.4%	—	—	—	—	12.1%	—	—	—	—	—	—	34.5%*
Number of studies	—	—	1	7	1	1	9	2	3	0	2	5	4	—	—	15
Study average	—	—	0.9%	6.4%	0.9%	5.0%	7.4%	3.5%	4.4%	0	7.3%	2.6%	2.5%	—	—	11.7%
Weighted average	—	—	0.9% (2/229)	4.3% (29/681)	0.9% (2/229)	5.0% (9/180)	5.9% (108/1831)	3.3% (4/121)	1.4% (10/721)	0	1.5% (10/654)	0.8% (8/973)	1.1% (15/1366)	—	—	8.6% (197/2288)

^*^Denotes imputed overall complication by adding up all AEs & ETEs. AE, adverse event; ETE, expected treatment effect; RF, radiofrequency.

For the combination procedures group, these studies typically involved lipoabdominoplasty, thighplasty, or brachioplasty, where helium plasma RF was applied as an adjunct to multimodal excisional surgery. Reported events primarily reflected known risks associated with the excisional components rather than the helium plasma RF treatment itself. Weighted average rates included 7.4% fluid accumulation events (seroma or hematoma), 5.4% wound healing complications or local tissue integrity (eg, necrosis, infection, dehiscence, open wound), 4.8% contour irregularities (eg, revision required, residual skin laxity, asymmetry), 3.6% pigmentary changes, 3.4% ophthalmologic or periorbital events (eg, blepharitis, conjunctival edema, ectropion, epiphora, lagophthalmos, photophobia, visual disturbances), 2.0% dermatological reactions (eg, contact dermatitis), 1.8% skin integrity and fibrotic tissue complications (eg, subcutaneous induration, burn, fibrosis, scarring), 0.9% cardiopulmonary and vascular complications (eg, anemia, hypoxemia, pulmonary embolism, superficial thrombophlebitis), and 0.5% gas-related/helium plasma-specific events (eg, gas buildup, bullae formation and subcutaneous emphysema, pneumoperitoneum) (Table 5).

**Table 5.** Systematic Review Adverse Event Rates and Weighted Rates—Multimodal Combination Procedures

			Percent of patients with an adverse event													
First author, year	Country	Sample size	Cardiopulmonary and vascular complications	Contour irregularities	Dermatological reactions	Edema/lymphatic complications	Fluid accumulation events	Gas-related/helium plasma-specific events	Neurologic effects/nerve impact	Ophthalmologic/periorbital events	Pigment changes	Skin integrity and fibrotic tissue complications	Wound healing complications/local tissue integrity	Article reported no AEs observed	Article reported only ETEs	Overall AE complication
Arshad et al (2023)^31^	USA	20	—	—	—	—	—	—	—	—	—	—	—	No AEs	—	0.0%
Barone et al (2025)^22^	Italy	38	—	—	—	—	5.3%	—	—	—	—	—	5.3%	—	—	10.5%*
Ruff et al (2022)^28^ AE	USA	192	—	2.1%	—	—	6.8%	0.5%	—	4.7%	—	1.6%	6.3%	—	—	24.0%
Ruff et al (2022)^28,42^ Gyn	USA	84	1.2%	—	—	—	7.1%	—	—	—	—	—	—	—	—	8.3%*
Ruff et al (2024)^27^	USA	100	—	—	2.0%	—	13.0%	—	—	1.0%	4.0%	2.0%	12.0%	—	—	34.0%*
Skenderian et al (2023)^45^	USA	115	—	—	—	—	—	—	—	—	1.7%	—	1.7%	—	—	3.5%*
Tambasco et al (2024)^49,52^ Lipoab	Italy	100	—	—	—	—	8.0%	—	—	—	8.0%	—	1.0%	—	—	17.0%*
Tambasco et al (2024)^49^ MaCh	Italy	226	0.4%	7.1%	—	—	4.9%	—	—	—	2.2%	—	0.9%	—	—	15.5%*
Tambasco et al (2025)^50,51^	Italy	21	—	—	—	—	4.8%	—	—	—	4.8%	—	4.8%	—	—	14.3%*
Vanek (2025)^29^	USA	40	2.5%	—	—	—	15.0%	—	—	—	—	2.5%	32.5%	—	—	52.5%*
Zorilla^30^ (2022)	USA	21	—	—	—	—	4.8%	—	—	—	—	—	4.8%	—	—	9.5%*
Number of studies	—	—	3	2	1	0	9	1	0	2	5	3	9	—	—	11
Study average	—	—	1.4%	4.6%	2.0%	0	7.7%	0.5%	0	2.9%	4.1%	2.0%	7.7%	—	—	17.2%
Weighted average	—	—	0.9% (3/350)	4.8% (20/418)	2.0% (2/100)	0	7.4% (61/822)	0.5% (1/192)	0	3.4% (10/292)	3.6% (20/562)	1.8% (6/332)	5.4% (46/853)	—	—	18.1% (173/957)

^*^Denotes imputed overall complication by adding up all AEs & ETEs. AE, adverse event; ETE, expected treatment effect.

### Efficacy Findings From Systematic Review

Several studies included objective or semiobjective indices to support efficacy assessment. Reported measures included Cutometer-based skin elasticity measurements (Courage + Khazaka electronic GmbH, Cologne, Germany), standardized circumferential measurements, and validated patient-reported outcome tools such as BODY-Q. In addition, multiple studies incorporated blinded IPR of standardized clinical photographs, providing a structured visual assessment method that reduces assessor bias compared with unblinded subjective evaluations. However, objective measurement tools were inconsistently applied across studies, limiting direct comparison and pooled interpretation.

Subjective improvements in skin laxity and contour were consistently reported by treating physicians, independent reviewers, and patients (Tables 1 and 2). While objective measurements of skin contraction were infrequently reported, many studies utilized GAIS assessments, IPR evaluations, and patient satisfaction surveys to support perceived improvements. Patient satisfaction ranged from 73%^12^ to 100%.^46-48,50^ GAIS rated by patients ranged from 67%^25^ to 100%,^46-49^ while ratings by investigators ranged from 79%^25^ to 100%.^46,47^ Several studies incorporated IPR evaluations, which closely aligned with patient-reported outcomes, strengthening the validity of the findings.^12,25,34,37,38,41,46-48,50^

Other reported performance outcomes included statistically significant improvements across BODY-Q scale scores,^22^ reductions in surface area measurements,^26,38,41,46^ and decreases in laxity test scores.^23,47^ Multiple authors highlighted that helium plasma RF enhances aesthetic outcomes across a broad range of anatomical areas, including regions prone to residual laxity such as the arms, back, thighs, and abdomen.^23,31-33,35,39,45,51^

### Meta-Analysis Results

#### Patient Satisfaction

**Figure 4.**
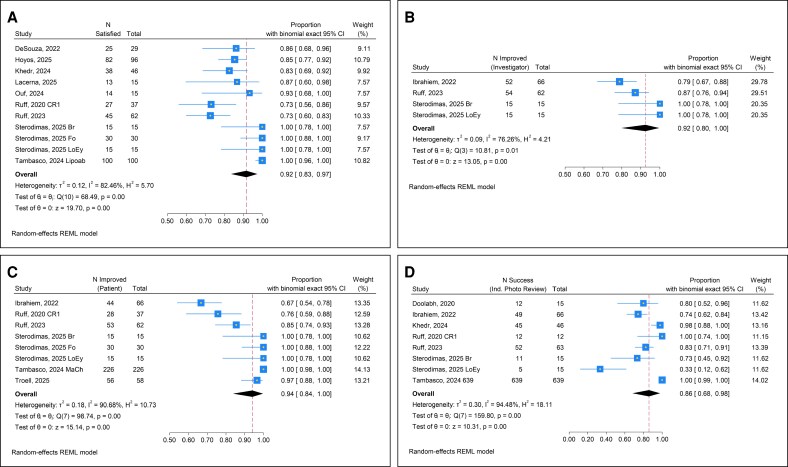
Meta-analysis of patient satisfaction and aesthetic improvement following helium plasma RF. Forest plots show pooled proportions with 95% CIs for (A) patient satisfaction (92%, 95% CI: 83%, 97%), (B) investigator-rated GAIS improvement (92%, 95% CI: 80%, 100%), (C) patient-rated GAIS improvement (94%, 95% CI: 84%, 100%), and (D) independent photographic review (IPR) improvement (86%, 95% CI: 68%, 98%).

The meta-analysis of patient satisfaction included 11 studies and 460 subjects. The pooled satisfaction rate was 92% (95% CI: 83%, 97%, *I*^2^: 82%) (Figure 4A), indicating a high overall level of patient-reported improvement. No evidence of publication bias was detected (Egger's test, *P* = .54).

**Figure 5.**
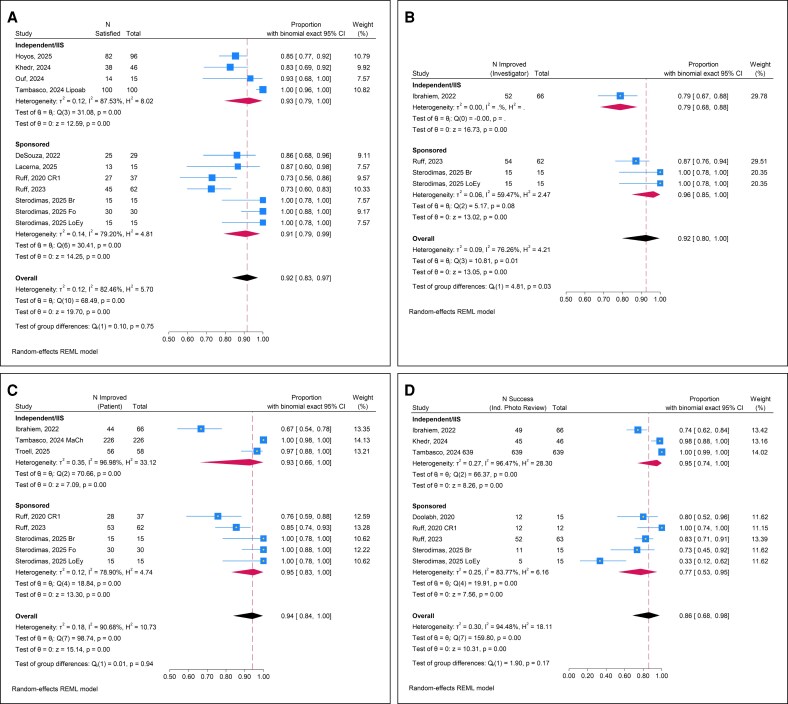
Subgroup analysis of outcomes by funding source. Forest plots compare pooled estimates between independent/investigator-initiated and industry-sponsored studies for (A) patient satisfaction (*P* = .75, not statistically significant), (B) investigator GAIS (*P* = .03, statistically significant; however, findings should be interpreted cautiously given the limited number of studies in this subgroup), (C) patient GAIS (*P* = .94, not statistically significant), and (D) IPR (*P* = .17, not statistically significant).

**Figure 6.**
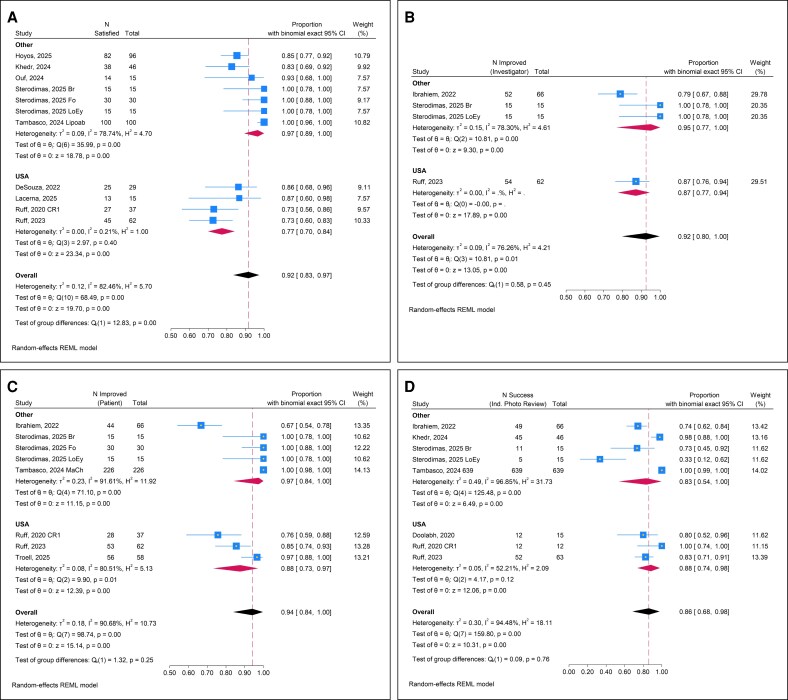
Subgroup analysis of outcomes by geographic region. Forest plots compare pooled estimates between U.S. and non-U.S. studies for (A) patient satisfaction (*P* < .001, statistically significant), (B) investigator GAIS (*P* = .45, not statistically significant), (C) patient GAIS (*P* = .25, not statistically significant), and (D) IPR (*P* = .76, not statistically significant).

Subgroup analysis by funding source showed comparable satisfaction rates between independent or investigator-initiated studies (93%, 95% CI: 79%, 100%, *I*^2^: 88%) and Apyx Medical-sponsored studies (91%, 95% CI: 79%, 99%; *I*^2^: 79%, *P* = .75) (Figure 5A). In contrast, subgroup analysis by geographical region demonstrated a statistically significant difference, with higher satisfaction rates in non-US studies (97%, 95% CI: 89%, 100%, *I*^2^: 79%) compared with US-based studies (77%, 95% CI: 70%, 84%; *I*^2^: 0.21%, *P* < .001) (Figure 6A).

**Figure 7.**
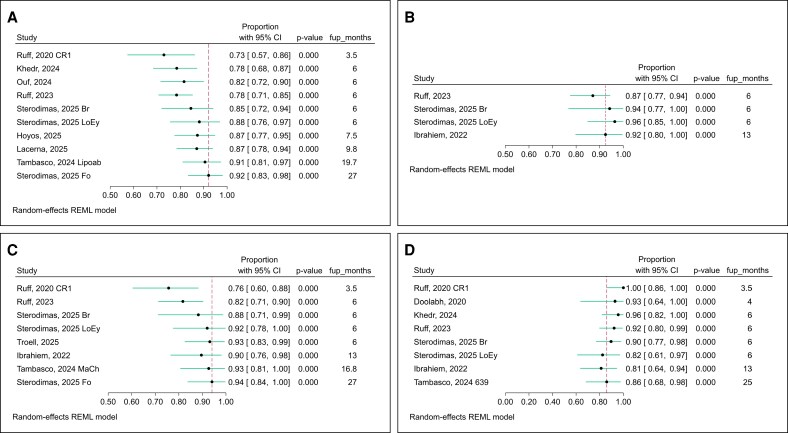
Meta-regression analysis of follow-up duration. Scatter plots with fitted regression lines illustrate the relationship between follow-up duration and outcomes for (A) patient satisfaction (*P* = .003, statistically significant), (B) investigator GAIS (*P* = .14, not statistically significant), (C) patient GAIS (*P* = .33, not statistically significant), and (D) IPR (*P* = .22, not statistically significant). REML, restricted maximum likelihood.

Meta-regression demonstrated a significant association between longer follow-up durations and higher satisfaction rates (*P* = .003), suggesting progressive improvement over time as remodeling and neocollagenesis occur (Figure 7A). Sensitivity analysis using a leave-one-out approach confirmed the robustness, with no single study disproportionately influencing the pooled estimate (Appendix).

#### Investigator Global Aesthetic Improvement Scale

Across the 4 studies and 158 subjects reporting Investigator GAIS, the pooled proportion of subjects rated as improved was 92% (95% CI: 80%, 100%, *I*^2^: 76%) (Figure 4B). A significant small-study effect was detected (Egger's test, *P* = .0020), indicating potential publication bias favoring studies with smaller sample sizes.

Additional results are shown in Figures 5B (subgroup analysis by sponsor), 6B (subgroup analysis by country), and 7B (follow-up duration). These findings should be interpreted cautiously given the limited number of studies included.

#### Patient Global Aesthetic Improvement Scale

Across the 8 studies and 509 subjects reporting patient GAIS, the pooled proportion of subjects reporting improvement was 94% (95% CI: 84%, 100%, *I*^2^: 91%) (Figure 4C), with no evidence of publication bias (Egger's test, *P* = .66).

Subgroup analysis by funding source showed similar rates between independent or investigator-initiated studies (93%, 95% CI: 66%, 100%, *I*^2^: 97%) and Apyx Medical-sponsored studies (95%, 95% CI: 83%, 100%), *I*^2^: 79%, *P* = .94 (Figure 5C). Subgroup analysis by country revealed slightly higher rates in non-US studies (97%, 95% CI: 84%, 100%, *I*^2^: 92%) compared with US-based studies (88%, 95% CI: 73%, 97%, *I*^2^: 81%), *P* = .25 (Figure 6C). Neither analysis reached statistical significance, indicating consistency in outcomes regardless of funding source or geographic location.

Meta-regression found no association between follow-up length and patient-reported improvement (*P* = .3252) (Figure 7C). Sensitivity analyses confirmed the stability of the pooled estimate across studies.

#### Independent Photographic Review

Independent photographic assessments were reported in 8 studies and 871 subjects, with a pooled improvement rate of 86% (95% CI: 68%, 98%, *I*^2^: 94%) (Figure 4D). No evidence of publication bias was detected (Egger's test, *P* = .1618).

Subgroup analysis by funding source revealed higher pooled IPR rates for independent or investigator-initiated studies (95%, 95% CI: 74%, 100%, *I*^2^: 96%) compared with Apyx Medical-sponsored studies (77%, 95% CI: 53%, 95%, *I*^2^: 84%), though this difference was not statistically significant (*P* = .17) (Figure 5D). Similarly, subgroup analysis by country showed comparable rates in non-US studies (83%, 95% CI: 54%, 100%, *I*^2^: 97%) and with US-based studies (88%, 95% CI: 74%, 98%; *I*^2^: 52%, *P* = .76) (Figure 6D). These findings indicate that neither geographic location nor funding source significantly impacts outcomes.

Meta-regression demonstrated no significant effect of follow-up length on IPR scores (*P* = .2198) (Figure 7D). Sensitivity analyses confirmed consistency of these findings across studies.

#### Complications

**Figure 8.**
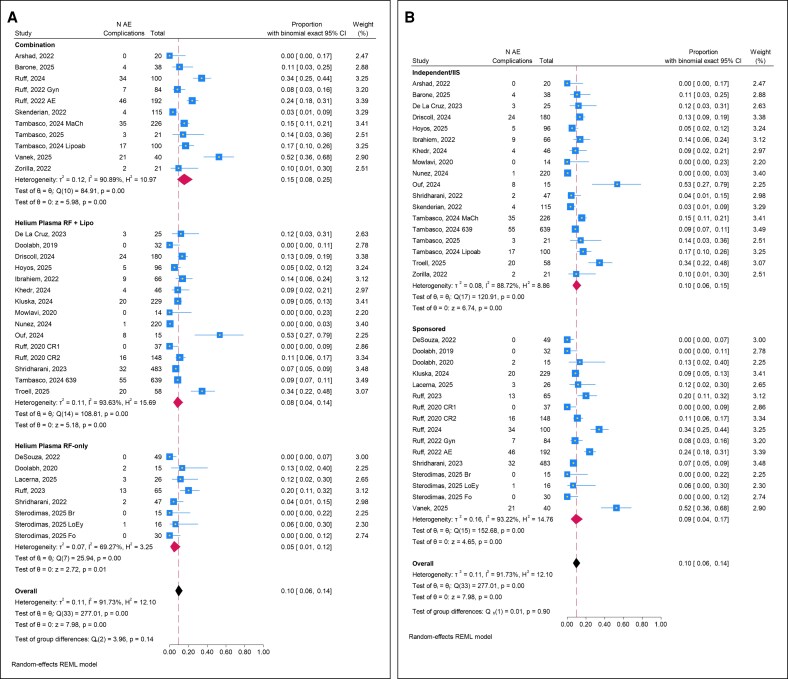

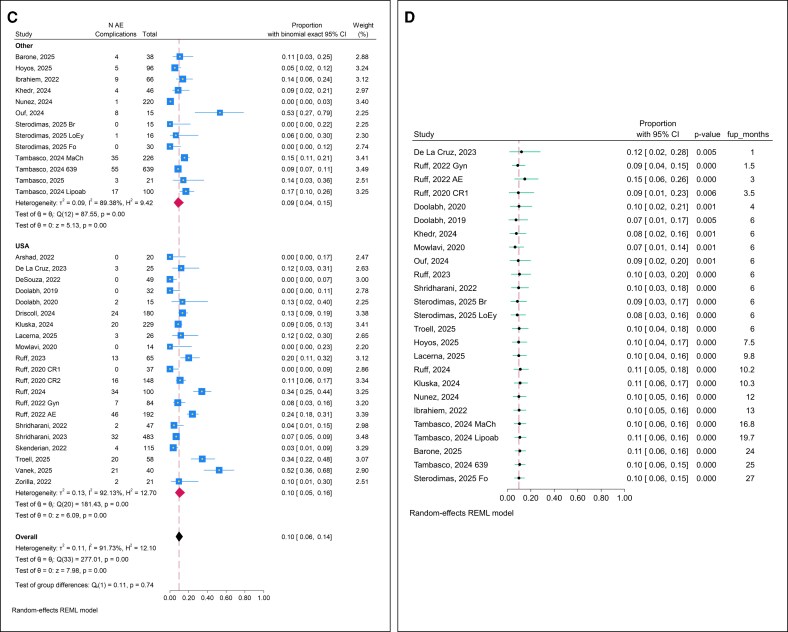
Meta-analysis of complication rates following helium plasma radiofrequency (RF). Forest plots show pooled AE rates by (A) procedural category, including helium plasma RF-only, helium plasma RF + liposuction, and combination procedures (test for subgroup differences *P* = .14, not statistically significant). Subgroup analyses demonstrate no significant differences in complication rates (B) by funding source (*P* = .90) or (C) geographic region (*P* = .74). (D) Meta-regression analysis shows no significant association between follow-up duration and complication rates (*P* = .82). REML, restricted maximum likelihood.

Subgroup meta-analysis of complication rates was conducted across 3 procedural categories: helium plasma RF-only subdermal applications, helium plasma RF + liposuction, and combination procedures (eg, helium plasma RF used with multimodal excisional surgery) (Figure 8A). The test for subgroup differences was not statistically significant (*P* = .14), indicating that AE rates did not differ meaningfully across procedure types.

For the helium plasma RF-only group (8 studies and 263 subjects), the pooled AE rate was 5% (95% CI: 1%, 12%; *I*^2^: 69%, *P* < .001), representing the lowest rate among procedural subgroups. For the helium plasma RF + liposuction group (15 studies and 2288 subjects), the pooled AE rate was 8% (95% CI: 4%, 14%; *I*^2^: 94%, *P* < .001). For the combination procedures group (11 studies and 957 subjects), the pooled AE rate was 15% (95% CI: 8%, 25%; *I*^2^: 91%, *P* < .001), the highest among the subgroups. Across all procedure types (34 studies and 3508 subjects), the overall pooled complication rate was 10% (95% CI: 6%, 14%, *I*^2^: 92%). No evidence of publication bias or small-study effects was detected (Egger's test, *P* = .8547), indicating stability of the results across both small and large studies.

Subgroup analysis by funding source demonstrated no statistically significant differences between independent/investigator-initiated studies (*P* = .90) and industry-sponsored studies (Figure 8B). Similarly, subgroup analysis by geographic region was also not statistically significant (*P* = .74) between U.S. and non-U.S. populations (Figure 8C).

Meta-regression by follow-up duration found no significant relationship between follow-up time and reported complication rates (*P* = .818, Figure 8D). Sensitivity analysis using a leave-one-out approach confirmed the robustness of the pooled complication rate, with no single study disproportionately influencing the overall results. Detailed statistical outputs and supporting analyses not included in the main text are provided in the Appendix.

## DISCUSSION

This systematic review and meta-analysis provide the most comprehensive evaluation to date of the safety and performance of helium plasma RF for aesthetic subdermal applications. As the demand for minimally invasive body contouring solutions continues to rise, it has become increasingly important to critically assess the safety, performance, and appropriate clinical use of emerging technologies. By consolidating data from more than 3500 treated subjects across 34 studies, this review clarifies the clinical profile of the helium plasma RF technology and highlights both its strengths and areas where further research is needed.

Across the evaluated studies, helium plasma RF was most commonly used in conjunction with liposuction procedures to enhance aesthetic outcomes in patients with mild to moderate skin laxity. Its precise subdermal energy delivery and minimal thermal spread were frequently cited as advantages, particularly in addressing residual laxity that liposuction alone may not fully correct.

Collectively, the findings of this systematic review and meta-analysis suggest a favorable safety and performance profile for helium plasma RF in aesthetic subdermal applications. High rates of patient satisfaction and investigator-rated improvement were consistently observed across diverse populations, treatment areas, and practice settings. While heterogeneity in study design, patient populations, and outcome measures contributed to variability in pooled estimates, sensitivity analyses confirmed the stability and robustness of these findings. Meta-regression identified a significant association between longer follow-up durations and higher patient satisfaction rates (*P* = .003), consistent with the progressive effects of tissue remodeling and neocollagenesis over time.

Subgroup analyses showed no statistically significant differences in performance outcomes between independent/investigator-initiated studies and industry-sponsored studies, supporting the external validity and reproducibility of the results. Satisfaction rates were higher in non-U.S. studies (97%; 95% CI: 89%-100%) compared with U.S. studies (77%; 95% CI: 70%-84%; *P* < .001), suggesting regional variation in patient-reported outcomes; however, outcomes remained favorable across all regions. Similarly, complication rates did not differ significantly by procedural type, funding source, or geographic region. Serious AEs were rare, and no unexpected safety concerns were identified.

Combination procedures involving helium plasma RF used as a component of large multimodal excisional surgeries (eg, abdominoplasty, brachioplasty, or thighplasty) were included in the literature but are outside the device's cleared indications. The system is cleared for the coagulation of subcutaneous soft tissue following liposuction for aesthetic body contouring, and findings from excisional procedures should not be generalized to routine use conditions.

By synthesizing data across diverse geographic regions, study designs, and sponsorship types, this analysis supports the consistency and generalizability of observed outcomes across clinical settings. Swanson et al^54^ previously published the first systematic review evaluating helium plasma RF for aesthetic applications, providing an early synthesis of the available literature. However, their review was limited in scope, including only a subset of available studies and lacking a quantitative analysis. The present systematic review builds upon this foundation by incorporating a robust search strategy and a concurrent meta-analysis to provide more precise estimates of safety and performance outcomes. This approach offers a more robust and clinically relevant evaluation of helium plasma RF across diverse treatment areas.

Helium plasma RF offers several advantages over other energy-based technologies. Its combined use of RF energy and helium plasma enables precise tissue targeting with minimal thermal spread while achieving temperatures required for collagen denaturation and immediate tissue contraction.^14^ The rapid cooling properties associated with helium plasma RF may also reduce the risk of overheating superficial layers and unintended thermal injury. Studies included in this review highlighted the ability to achieve predictable contraction with less complications compared to bulk-heating RF devices.^26^ Although direct comparative data remain limited, the available evidence demonstrates a favorable safety profile and consistently reported aesthetic improvements with helium plasma RF across anatomical areas.^23-26^

The pooled analysis confirmed that helium plasma RF is a safe, minimally invasive treatment option for patients with mild to moderate skin laxity. Most AEs were minor and self-limited, resolving with conservative management.

Weighted analysis of data across studies demonstrated low rates of clinically significant AEs, with the highest incidence occurring in fluid accumulation events (hematoma and seroma). Seroma is a known risk of ultrasound-assisted liposuction with average rates ranging from 0% to 29.9% in studies with up to 1772 patients.^55-60^ Wound healing event rates were higher in studies involving excisional procedures, such as lipoabdominoplasty, thighplasty, or brachioplasty, where helium plasma RF was used as a part of the overall surgical approach. These events are considered common risks of the excisional procedures themselves rather than the helium plasma RF treatment.^5^

The overall pooled complication rate of 10% reflects a generally favorable safety profile. Although the absolute AE rate for standalone helium plasma RF (5%) was numerically lower than that of helium plasma RF combined with liposuction (8%) or combination excisional procedures (15%), these differences were not statistically significant. This suggests that, when used appropriately and within cleared indications, helium plasma RF does not meaningfully increase procedural risk, even when used adjunctively with liposuction.

Caution is warranted when interpreting AE rates in studies that include major excisional surgeries. The higher pooled rate in this subgroup likely reflects the intrinsic risks of procedures such as abdominoplasty and brachioplasty, rather than risks specific to helium plasma RF. Because excisional procedures fall outside the current cleared indications for helium plasma RF, results from these studies should not be generalized to routine device use.

Serious AEs such as skin necrosis, thromboembolism, or subcutaneous emphysema were either absent or rare, and no mortality was reported. These results are consistent with prior retrospective and prospective reports, including high-volume studies demonstrating that adjunctive use of helium plasma RF during liposuction is comparable to other minimally invasive aesthetic procedures.^7,8,26,43^ Collectively, these data support the safe integration of helium plasma RF into routine aesthetic practice when used according to appropriate protocols.

Performance outcomes from this review highlight the clinical utility of helium plasma RF as both a standalone procedure and as an adjunct to liposuction. Across studies, treating physicians, independent reviewers, and patients consistently reported subjective and photographic improvements in skin laxity and body contour. The meta-analysis quantified these outcomes, demonstrating a pooled patient satisfaction rate of 92%, with meta-regression showing that longer follow-up durations were associated with higher satisfaction rates (*P* = .003), suggesting progressive improvement over time as tissue remodeling and neocollagenesis continue beyond the initial treatment period.

This progressive remodeling has also been reported with other thermal-based technologies, reinforcing that both clinical- and patient-perceived benefits may continue to evolve for months following treatment. Improvements measured by the Investigator and Patient GAIS and IPR also supported the effectiveness of the treatment. While heterogeneity was present across studies, likely due to differences in study design, patient populations, and outcome measurement tools, sensitivity analyses confirmed the robustness of the pooled estimates.

Most of the included studies evaluated helium plasma RF in combination with liposuction, where its unique mechanism of action, targeting the FSN with precise subdermal energy delivery, addresses residual laxity that liposuction alone cannot correct. Several studies reported enhanced contouring and superior aesthetic outcomes with the addition of helium plasma RF without increasing complication rates.^25,28,29,43^ Authors consistently report positive performance outcomes with helium plasma RF following liposuction. These findings support the integration of helium plasma RF as an adjunctive tool to optimize body contouring results in appropriately selected patients.

Subgroup analyses by funding source demonstrated no statistically significant differences in performance outcomes between independent or investigator-initiated studies and those sponsored by Apyx Medical, supporting the external validity of the findings. However, subgroup analysis by geographic region revealed significantly higher satisfaction rates in non-U.S. studies compared with U.S. studies (*P* < .001), though satisfaction remained high across all regions. Cultural variation in patient expectations, differences in procedural protocols, and variation in follow-up duration may have contributed to these regional differences. This discrepancy did not extend to investigator-rated improvement or IPR scores, suggesting consistent physician-evaluated aesthetic outcomes globally.

Independent photographic review scores were slightly lower than subjective measures such as patient or investigator GAIS, which is not unexpected given the conservative nature of independent photographic evaluations and inherent limitations of 2-dimensional imaging in capturing 3-dimensional or tactile improvements.

Overall, the evidence synthesized in this review suggests that helium plasma RF is well suited for patients with mild to moderate skin laxity seeking minimally invasive contour improvement. It may fill an important gap between liposuction alone and excisional procedures.

This review has several strengths, including a comprehensive literature search, adherence to PRISMA methodology, and the integration of both qualitative systematic review and quantitative meta-analysis. The use of standardized risk-of-bias tools and sensitivity analyses enhances the reliability of the conclusions. However, limitations must be considered. Although a comprehensive literature search was performed and prespecified inclusion criteria were applied, screening and full-text eligibility assessment were conducted by a single reviewer, which may increase the risk of selection bias compared with dual independent screening. Similarly, data extraction was performed by a single reviewer, which may increase the risk of transcription error or subjective interpretation of outcomes. The included studies were heterogeneous with respect to study design, treated anatomical areas, procedural techniques, outcome measures, and follow-up duration, contributing to substantial statistical heterogeneity in several pooled estimates. A formal certainty-of-evidence assessment was not performed; therefore, overall confidence in pooled estimates should be interpreted in the context of heterogeneity and the predominance of observational study designs.

Most included studies were Level IV case series or retrospective cohort analyses, limiting causal inference and preventing quantification of the incremental contribution of helium plasma RF relative to liposuction alone. Consistent with this, risk-of-bias assessment demonstrated that the majority of nonrandomized studies were at moderate risk of bias, with approximately one-third classified as having serious risk of bias, primarily due to retrospective design, lack of control groups, absence of blinding, and reliance on subjective outcome measures.

Objective indices reported in selected studies included Cutometer-based elasticity testing, circumferential measurements, validated patient-reported outcome instruments (eg, BODY-Q), and blinded IPR; however, these measures were inconsistently applied across the evidence base. Many performance outcomes relied on subjective, nonstandardized assessment tools (eg, GAIS and patient satisfaction), further limiting comparability across studies. Although a large cumulative number of patients has been reported, the evidence base remains largely observational and heterogeneous; therefore, cumulative sample size supports consistency of reported outcomes but should not be interpreted as definitive proof of efficacy. Prospective controlled trials using standardized, validated outcome measures and longer follow-up are needed to more conclusively define treatment effect. Finally, because the literature includes studies where helium plasma RF was used in combination with procedures outside current cleared indications, findings from these subgroups should be interpreted cautiously and should not be generalized to routine use conditions.

Despite these limitations, the findings remain clinically meaningful and reflective of real-world practice. In aesthetic surgery, high-quality randomized data are often limited, and much of the evidence base is derived from observational and retrospective studies. The consistency of outcomes across a large cumulative dataset, multiple study designs, and diverse clinical settings supports the reliability and external validity of the observed safety profile and performance trends.

This systematic review and meta-analysis therefore contribute meaningfully to the existing knowledge base by providing the most comprehensive evaluation to date of helium plasma RF, both as a stand-alone treatment and adjunct to liposuction. This broader scope helps fill a critical gap in the literature and offers valuable insights into how helium plasma RF may be incorporated into aesthetic body contouring procedures and integrated into routine aesthetic surgical practice. Moreover, the findings enhance the understanding of treatment outcomes and safety profiles across diverse patient populations and practice settings, supporting evidence-based decision-making for clinicians.

Importantly, the cumulative evidence synthesized in this review adds value beyond individual case series by providing the largest consolidated assessment of helium plasma RF outcomes to date. Aggregating data from more than 3500-treated subjects enables more precise estimation of complication rates and summary performance outcomes and allows evaluation of consistency across anatomical regions, geographic settings, and sponsorship types. Although the literature remains predominantly observational, the pooled dataset strengthens confidence in the reproducibility of reported safety findings and the consistency of patient- and investigator-reported improvement across studies. The widespread adoption of this technology and the remaining gaps in the literature highlight the need for future research, including randomized controlled trials comparing helium plasma RF with liposuction alone or other minimally invasive devices, standardized patient-reported outcome tools such as BODY-Q, and extended follow-up periods (>24-48 months) to better evaluate durability of outcomes.

## CONCLUSIONS

This systematic review and meta-analysis provide the most comprehensive evaluation to date of the safety and performance of helium plasma RF for aesthetic subdermal applications. Across 33 peer-reviewed publications representing 34 distinct studies and encompassing 3508 treated patients, helium plasma RF demonstrated a consistently favorable safety profile. Major AEs were rare, and most reported complications were minor and self-limited, resolving spontaneously or with conservative management.

The meta-analysis further quantified these findings, demonstrating a high pooled patient satisfaction rate of 92% and consistent improvements in Investigator GAIS, patient GAIS, and IPR scores. Meta-regression demonstrated that longer follow-up was significantly associated with higher satisfaction rates, reflecting the progressive nature of tissue remodeling and neocollagenesis over time. Subgroup analyses revealed no statistically significant differences between independent or investigator-initiated studies and industry-sponsored studies, supporting the absence of sponsorship bias. Satisfaction rates were significantly higher in non-U.S. studies compared with U.S. studies, though outcomes remained favorable across all regions, supporting consistency of findings across regions.

## Supplemental material

This article contains supplemental material located online at https://doi.org/10.1093/asjof/ojag092.

## Supplementary Material

ojag092_Supplementary_Data
