# Supplementary material for: Helium Plasma Radiofrequency for Aesthetic Subdermal Treatment: A Systematic Review and Meta-Analysis
Source: Aesthet Surg J Open Forum. 2026 May 22;8:ojag092. doi: 10.1093/asjof/ojag092 (PMC13293070; doi:10.1093/asjof/ojag092)
Supplement: ojag092_Supplementary_Data [file ojag092_supplementary_data.docx]

**Appendix**

**Search Strategy**

Search terms are constructed to capture all relevant references related to Renuvion technology. Boolean operators (e.g., AND, OR) and term variations (e.g., synonyms and alternative spellings) will be used to enhance search sensitivity and specificity.

This search protocol follows the principles of the [*Cochrane Handbook for Systematic Reviews of Interventions*](https://www.cochranelibrary.com/about/about-cochrane-reviews), which emphasizes transparency, reproducibility, and methodological rigor in literature reviews.

Search terms were selected based on the device label name and its known synonyms, manufacturer, and intended clinical applications. The strategy accounts for variations in device naming (e.g., Renuvion, J-Plasma) and terminology used across international publications.

Search strings were iteratively generated, tested, and optimized across selected databases. Each string is tailored to the unique syntax and capabilities of the database to maximize retrieval of pertinent results. The finalized protocol is reviewed by a medical expert to ensure the clinical relevance and medical accuracy of the search terms, with revisions made as needed based on subject-matter expertise.

No date limits were applied to the initial searches to capture all available evidence. Additional terms may be introduced during the review process if new, relevant terminology is identified.

**Search Strings**

PubMed/Europe PMC/Science Direct: ("Renuvion" OR "J-Plasma" OR "JPlasma" OR "helium plasma") AND (“Apyx”)

Google Scholar *(Articles, not Case Law)*: ("Renuvion" OR "J-Plasma" OR “JPlasma” or “helium plasma" OR "radiofrequency plasma") AND ("safety" OR "efficacy" OR “effectiveness” OR "outcomes") AND (“Apyx”)

Society Website Keywords *(The Aesthetic Society, American Academy of Cosmetic Surgery, American Society of Plastic Surgeons, International Society of Aesthetic Plastic Surgery, American Academy of Dermatology) -* Use internal site search tools with combinations like: “Renuvion”, “Helium Plasma”, “J-Plasma”

**Full-Text References Excluded and Reasons for Exclusion**

39 references were identified through targeted searches of professional society websites:

- ASAPS: 4
- American Academy of Cosmetic Surgery: 8
- American Association of Plastic Surgeons: 7
- ASPS: 16
- AAD (search “Renuvion” only): 4

However, all 39 were excluded because they did not contain relevant clinical data.

In addition, the following references were reviewed and excluded:

| **Citation** | **Full Review Outcome** |
| --- | --- |
| DeLozier JB, Holcomb JD. Helium Plasma Dermal Resurfacing With and Without Concurrent Aesthetic Surgery of the Face and Neck: A Retrospective Review, Aesthetic Surgery Journal Open Forum, 2022;, ojac061, https://doi.org/10.1093/asjof/ojac061 | Exclude – Dermal Resurfacing |
| Holcomb JD, Doolabh V, Lin M, Zimmerman E. High energy, double pass helium plasma dermal resurfacing: A prospective, multicenter, single-arm clinical study. Lasers Surg Med. 2022 Feb 16. doi: 10.1002/lsm.23524. Epub ahead of print. PMID: 35170772. | Exclude – Dermal Resurfacing |
| Holcomb JD, Kelly M, Hamilton TK, and DeLozier JB. A Prospective Study Evaluating the Use of Helium Plasma for Dermal Resurfacing. Lasers in Surgery and Medicine, 2020 Apr 29. DOI: 10.1002/lsm.23257. | Exclude – Dermal Resurfacing |
| Holcomb JD. Helium plasma dermal resurfacing: VISIA CR assessment of facial spots, pores, and wrinkles – Preliminary findings. JCD 26 Mar 2021. https://doi.org/10.1111/jocd.14106. | Exclude – Dermal Resurfacing |
| Leake D, Lee J. Applications of Helium Plasma in Rejuvenation of the Face and Neck [Internet]. Enhanced Liposuction - New Perspectives and Techniques. IntechOpen; 2022. Available from: http://dx.doi.org/10.5772/intechopen.100162 | Exclude – Dermal Resurfacing |
| Hoyos AE, Perez ME, Mogollon IR, Arcila A. Decision-Making Algorithm for Advanced Excisional Body Contouring: Dynamic Definition Solutions for Skin Laxity. Plast Reconstr Surg. 2022 Dec 1;150(6):1248-1259. doi: 10.1097/PRS.0000000000009740. Epub 2022 Sep 19. PMID: 36112807. | Exclude - Case Report >10 |
| Vasilakis V, Isakson MH, Yamin F, Kortesis BG, Bharti G, Hunstad JP. Four-Position Four-Entry Site Circumferential Arm Liposuction: Technique Overview and Experience. Aesthetic Plast Surg. 2020 Oct;44(5):1596-1603. doi: 10.1007/s00266-020-01857-1. Epub 2020 Jul 8. PMID: 32642814. | Exclude - Case Report >10 |
| Levy A, Berl A, Shir-Az O, Mann D, Weiss E, Shalom A. Abdominal Etching-A Novel Classification Method for Surgical Approach. Aesthetic Plast Surg. 2025 Jan;49(2):538-546. doi: 10.1007/s00266-024-04384-5. Epub 2024 Oct 1. PMID: 39354230; PMCID: PMC11813945. | Exclude - Data is not on the Target Technology |
| Tambasco D, Hoyos A, Perez M, Tomaselli F, Mir J, Albanese R. Optimizing Safety and Precision for Secondary Liposuctions: Integrating Intraoperative Ultrasound With VASER Technology. Aesthet Surg J. 2025 Jan 16;45(2):NP57-NP64. doi: 10.1093/asj/sjae213. PMID: 39412204. | Exclude - Data is not on the Target Technology |
| Troell RJ. Lipoabdominoplasty: Comparing Ultrasound-Assisted and Power-Assisted Techniques. The American Journal of Cosmetic Surgery. 2022;40(4):279-292. doi:10.1177/07488068221099153 | Exclude - Data is not on the Target Technology |
| Danilla S, Abou-Kheir N, Gonzalez R, Richer D. The Fourth-Generation Lower Body Lift: Combining Techniques and Technologies for Improved Outcomes in High-Demand Female Patients. Aesthetic Plast Surg. 2025 Apr 21. doi: 10.1007/s00266-025-04877-x. Epub ahead of print. PMID: 40259068. | Exclude - Mixed device & procedure data without Renuvion data stratified |
| Troell RJ. Aesthetic Primary and Secondary Breast Augmentation: Proposed Algorithm for Optimal Cosmetic Outcomes While Minimizing Complications. The American Journal of Cosmetic Surgery. 2025;0(0). doi:10.1177/07488068251314535 | Exclude - Mixed device & procedure data without Renuvion data stratified |
| Gueli Alletti S, Rosati A, Capozzi VA, et al. Use of laparoscopic and laparotomic J-Plasma handpiece in gynecological malignancies: results from a pilot study in a tertiary care center. Front Oncol. 2022;12:868930. doi:10.3389/fonc.2022.868930. | Exclude – Application Surgical, not Aesthetic Subdermal |
| Iavazzo C, Fotiou A, Kokkali K, Vorgias G. Experience With J-Plasma Device in Achieving Complete Cytoreduction in Patients With Ovarian Cancer. Cancer Diagn Progn. 2023 May 3;3(3):392-397. doi: 10.21873/cdp.10230. PMID: 37168972; PMCID: PMC10165381. | Exclude – Application Surgical, not Aesthetic Subdermal |
| Manuel M. Laparoscopic Treatment of Benign and Malignant Pelvic Disorders Utilizing Helium Plasma: A Retrospective Analysis. BioMedical Journal of Scientific & Technical Research. December 23, 2020. DOI: 10.26717/BJSTR.2020.32.005310. | Exclude – Application Surgical, not Aesthetic Subdermal |
| Moncada S, Nichols C. Helium Plasma-Assisted Breast Reduction: A Pilot Study, Aesthetic Surgery Journal Open Forum, Volume 4, 2022, ojac041, https://doi.org/10.1093/asjof/ojac041 | Exclude – Application Surgical, not Aesthetic Subdermal |
| Mattei B, Colletti G, Negrello S, Anesi A, Sanna G, Nocini R. Treating head and neck venous malformations with cold helium plasma electrosurgical device: A 17 patients case series. J Craniomaxillofac Surg. 2025 Jun;53(6):773-779. doi: 10.1016/j.jcms.2025.02.024. Epub 2025 Feb 22. PMID: 39988533. | Exclude – Application Surgical, not Aesthetic Subdermal |
| Theodoulidis V, Prodromidou A, Stamatakis E, Alexakis N, Rodolakis A and Haidopoulos D: Application of J‑Plasma in the excision of diaphragmatic lesions as part of complete cytoreduction in patients with advanced ovarian cancer. Mol Clin Oncol 17: 113, 2022. | Exclude – Application Surgical, not Aesthetic Subdermal |

**Bias Appraisal Tables**

Two randomized controlled subdermal and aesthetic trials were evaluated using the Cochrane RoB 2 tool (see **Supplemental Table 1**). The study by Barone (2025) demonstrated low risk of bias across all domains, supported by appropriate randomization, consistent adherence to protocol, high follow-up rates, use of validated and blinded outcome assessments, and comprehensive reporting of prespecified endpoints. The study by Ouf (2024) was rated as having some concerns overall. Although it employed adequate randomization and reported complete data, the lack of blinding and limited clarity around potential protocol deviations and outcome measurement methods introduced uncertainty regarding potential bias.

The majority of subdermal and aesthetic studies included in this review were evaluated using the ROBINS-I tool (see **Supplemental Table 2**) and were found to have a moderate overall risk of bias, largely due to their retrospective, non-randomized designs, lack of control groups, and reliance on subjective outcome measures without blinding or validated assessment tools. While most studies clearly described the intervention and followed consistent protocols without significant missing data, key limitations included insufficient control for confounding variables (e.g., BMI, age, baseline skin laxity), potential selection bias, and limited statistical adjustment. A smaller subset of studies demonstrated serious risk of bias, primarily due to a combination of uncontrolled confounding, subjective outcome evaluation, and absence of comparator groups. Only a few studies achieved low risk across all domains, typically when prospectively designed, using standardized objective outcome measures and comprehensive reporting.

Supplemental Table 1: COCHRANE ROB2 ASSESSMENT FOR RANDOMIZED CONTROLED TRIALS ONLY

| **Reference** | **Bias arising from the randomization process** | **Bias due to deviations from intended interventions** | **Bias due to missing outcome data** | **Bias in measurement of the outcome** | **Bias in selection of the reported result** | **Overall Risk of Bias** |
| --- | --- | --- | --- | --- | --- | --- |
| Barone, 2025 (Italy)^1^ | Low Risk. Patients were randomly assigned, and baseline characteristics were comparable between groups. | Low Risk. Protocol appears to have been followed consistently across both groups. | Low Risk. 100% follow-up at 6 months; 84% at 2 years is acceptable for aesthetic studies. | Low Risk. Outcomes were assessed using validated BODY-Q and VAS scales, including blinded physician raters. | Low Risk. All prespecified outcomes appear to have been reported in full. | **Low Risk.** This RCT employed strong methodology: proper randomization, validated outcomes, blinded assessments, and high follow-up rates, supporting a robust evidence base. |
| Ouf, 2024 (Egypt)^2^ | Low Risk. Patients were randomly assigned using a sealed envelope method, and group sizes were balanced (n=15 per arm). Baseline characteristics appear similar, suggesting random sequence generation and allocation concealment were adequate. | Low Risk. Patients were not blinded, which introduces the possibility of behavior or response bias (e.g., reporting of pain, satisfaction). There’s no evidence of deviations from protocol, but lack of blinding in an unmasked study warrants caution. | Low Risk. Data were reported for all 45 randomized patients. There is no evidence of attrition or selective exclusion. | Moderate Risk. Outcomes include subjective measures (e.g., satisfaction, pain scores, symmetry), and no mention of blinded assessors is provided. This introduces potential detection bias. | Low Risk. All pre-specified outcomes (complications, satisfaction, volume reduction, skin redundancy, objective firmness via Cutometer) were reported. | **Moderate Risk.**  While the study design included proper randomization and complete data reporting, the lack of participant and assessor blinding for subjective outcomes results in some concerns in two domains, leading to a moderate overall risk of bias. |

Supplemental Table 2: ROBINS-I ASSESSMENT FOR NON-RANDOMIZED STUDIES AND CASE SERIES

| **Reference** | **Confounding** | **Selection of Participants** | **Classification of Interventions** | **Deviations from Intended Interventions** | **Missing Data** | **Measurement of Outcomes** | **Selection of Reported Results** | **Overall ROBINS-I Rating** |
| --- | --- | --- | --- | --- | --- | --- | --- | --- |
| Arshad, 2022 (USA)^3^ | Moderate risk. No adjustment was made for potential confounders such as BMI variation, comorbidities, extent of liposuction, or fat grafting technique. | Low risk. Inclusion was clearly defined: post-weight-loss patients undergoing back contouring with BBL. | Moderate risk. All patients received Renuvion, but procedural details varied and were individualized. | Moderate risk. No blinding of personnel or participants was reported, which could introduce performance bias. | Low risk. Outcomes were reported for all patients included. | Moderate risk. Outcomes were largely qualitative (e.g., satisfaction, contour improvements) without standardized or validated assessment tools. | Moderate risk. No predefined primary outcomes or statistical comparisons were reported. The study relied on narrative findings. | **Moderate Risk.** This single-arm retrospective study provides observational data with consistent reporting across patients, but its methodological limitations—absence of a control group, lack of validated outcomes, and individualized techniques—limit the strength of conclusions. |
| De La Cruz, 2023 (USA)^4^ | Moderate Risk. No statistical adjustment for BMI, age, comorbidities, or baseline laxity. Group assignment was not randomized, and variation in patient characteristics may influence outcomes. | Moderate Risk. Retrospective inclusion from a single surgeon’s practice without clearly defined selection criteria introduces potential selection bias. | Low Risk. Interventions were clearly defined (UAL-only vs. SEAL technique using UAL + PAL + Renuvion). | Low Risk. All patients received the intended treatment without reported protocol deviations. | Low Risk. Outcome data appear complete; no losses to follow-up were reported. | Moderate Risk. Safety outcomes were objective, but no standardized efficacy assessments were used; aesthetic outcomes were described qualitatively without validated scales or blinding. | Low Risk. All reported complications and procedural outcomes appear to have been disclosed; no evidence of selective outcome reporting. | **Moderate Risk.** The inclusion of a control group strengthens the design, but the absence of randomization, lack of adjustment for confounders, and use of subjective, non-blinded efficacy assessments limit internal validity. |
| DeSouza, 2022 (USA)^5^ | Moderate Risk. No statistical adjustment for potential confounders such as BMI, age, or liposuction extent. However, all patients received the same core procedure. | Moderate Risk. All patient charts were reviewed, but PSQ completion was voluntary, limiting generalizability of satisfaction data and introducing selection bias for subjective outcomes. | Low Risk. Intervention was clearly defined and consistently described across patients. No misclassification evident. | Low Risk. Standardized treatment parameters were used; no reported protocol deviations. | Moderate Risk. 29 of 49 patients completed the PSQ. While safety data appear complete, the missing satisfaction data may bias interpretation of efficacy. | Serious Risk. Satisfaction outcomes were subjective, collected via telephone, without use of validated tools or blinding. No objective efficacy endpoints were assessed. | Moderate Risk. Only descriptive outcomes were provided. No pre-specified analysis plan; reporting emphasized favorable satisfaction and safety findings. | **Serious Risk.** The retrospective design, absence of a comparator group, lack of statistical control for confounding, missing subjective outcome data, and reliance on non-validated satisfaction surveys without blinding collectively indicate a serious risk of bias. Objective efficacy data were not collected. |
| Doolabh, 2019 (USA)^6^ | Moderate Risk. No adjustment for patient-specific variables (e.g., BMI, age, baseline laxity), which may influence observed outcomes. | Moderate Risk. All patients were selected from a single surgeon's practice, without random sampling. | Low Risk. Intervention was clearly defined as adjunctive Renuvion after liposuction, and use was consistent across cases. | Low Risk. No deviations were reported; device settings and procedural approach were standardized. | Low Risk. Follow-up data appear complete; no missing outcome data were noted. | Moderate Risk. Outcomes were subjectively assessed without use of validated scales or blinded review. No quantitative measures of efficacy were reported. | Moderate Risk. Findings were presented descriptively without statistical analysis or pre-specified endpoints. Positive outcomes may be selectively emphasized. | **Moderate Risk.** While the study consistently applied a defined intervention and reported complete follow-up, the lack of objective measures, validated tools, blinding, and statistical analysis introduces a moderate risk of bias, limiting strength of inference. |
| Doolabh, 2020 (USA)^7^ | Moderate Risk. No control for age, BMI, or baseline skin laxity; potential influence from other body-area procedures. | Moderate Risk. Patients were selected based on retrospective chart availability without randomization or pre-specified inclusion/exclusion criteria. | Low Risk. Intervention (Renuvion subdermal neck coagulation) was clearly defined and consistently applied. | Low Risk. No protocol deviations reported; all treatments followed the intended technique. | Moderate Risk. One patient lost to follow-up and another excluded due to photo inconsistency; incomplete image-based outcomes. | Moderate Risk. Subjective image review combined with quantitative assessment, but lack of inter-rater reliability testing introduces some subjectivity. | Low Risk. Results appear comprehensive and include both safety and efficacy endpoints. | **Moderate Risk**. Study design limits internal validity, but data collection and reporting were reasonably thorough. |
| Driscoll, 2024 (USA)^8^ | Moderate Risk. Patient-level confounders such as BMI, hypertension, number of anatomic areas treated, and use of adjunctive VASER were not controlled for. No multivariate analysis was performed to isolate Renuvion-specific risks. | Moderate Risk. Retrospective single-site review with undefined inclusion/exclusion criteria may introduce selection bias. | Low Risk. Renuvion treatment was clearly described and consistently applied, with or without adjunctive VASER. | Low Risk. No protocol deviations were noted; treatment delivery aligned with intended methods. | Low Risk. Complications were reported for the entire cohort (n=180); no indication of missing outcome data. | Moderate Risk. Safety outcomes were assessed via retrospective chart review without standardized grading or blinded adjudication. No validated aesthetic or satisfaction measures were used. | Moderate Risk. The study did not specify a primary endpoint or analysis plan. Emphasis was placed on complication rates without formal statistical comparisons or outcome stratification. | **Moderate Risk.** This study provides useful observational safety data from a large, real-world cohort. However, its retrospective design, absence of control or adjustment for confounding variables (e.g., VASER use), and reliance on subjective outcome descriptions limit the internal validity and generalizability of efficacy conclusions. |
| Hoyos, 2025 (Columbia)^9^ | Moderate Risk. No control for key confounders such as BMI, age, or baseline laxity. Outcomes were not stratified by HDL-only vs. HDL + ancillary procedures. | Moderate Risk. Patients were consecutively treated at a single private center, but no detailed inclusion/exclusion criteria were provided. | Low Risk. All patients were treated with Renuvion + HDL using defined procedural steps. | Low Risk. No deviations from the treatment protocol were reported. | Moderate Risk. Satisfaction data only collected in 100 of 174 patients. | Moderate Risk. Outcomes relied on patient-reported satisfaction without validated scales. No objective aesthetic or skin tightening assessments were used. | Moderate Risk. Only descriptive statistics were reported; no subgroup analysis or inferential comparisons were performed. | **Moderate Risk of Bias.** This study provides useful descriptive data on safety and patient satisfaction following HDL + Renuvion. However, the retrospective design, lack of objective or stratified outcomes, and incomplete survey follow-up introduce moderate bias and limit causal interpretation. |
| Ibrahiem, 2022 (Egypt)^10^ | Moderate Risk. No stratification or control for key confounders such as arm laxity grade, BMI, or Fitzpatrick skin type. These variables may influence both aesthetic outcomes and complication rates. | Moderate Risk. Assignment was based on hospital availability and financial preference. While not influenced by clinical criteria, this pseudo-randomization still allows for systematic differences across groups. | Low Risk. Each group’s treatment modality was clearly defined and consistently applied (VASER + RFAL, VASER + Renuvion, VASER alone. | Low Risk. There were no reported deviations from planned interventions. All patients received the procedures as described. | Low Risk. There was no significant loss to follow-up, and all patients were included in the outcome reporting. | Moderate Risk. Outcomes were based on unblinded ratings from patients, independent surgeons, and the surgical team using subjective 4-point scales. No validated measurement tools or objective endpoints were used. | Low Risk. Full reporting of complication rates and aesthetic outcomes was provided across all groups. No evidence of selective outcome reporting. | Moderate Risk.  This study offers comparative insight across three energy-based lipoplasty groups but is limited by its non-randomized design, potential for uncontrolled confounding, and reliance on subjective unblinded outcome assessments. Nevertheless, full outcome reporting and consistent procedural execution strengthen internal consistency. |
| Khedr, 2024 (Egypt)^11^ | Moderate Risk. No statistical adjustment or stratification for age, BMI, or baseline skin laxity—factors that may influence both aesthetic outcomes and complication risk. | Moderate Risk. Consecutive patients were not clearly described, and selection criteria may have introduced bias due to lack of random sampling. | Low Risk. The intervention (liposuction + Renuvion) was clearly defined and consistently applied to all participants. | Low Risk. No deviations were reported; all treatments followed the intended surgical protocol. | Low Risk. All 46 patients were accounted for in both safety and satisfaction reporting; no evidence of loss to follow-up. | Moderate Risk. Outcomes were assessed using unblinded patient and surgeon satisfaction scales without validated tools or objective measures, increasing detection bias. | Moderate Risk. Only positive satisfaction categories were reported (e.g., “satisfied,” “excellent”), with limited transparency about how ratings were collected or interpreted. No statistical analysis was presented. | **Moderate Risk.** While the study provides real-world insight into safety and satisfaction with liposuction + Renuvion treatment, it is limited by lack of confounder control, unclear enrollment methods, unblinded and subjective outcome measures, and potential selective reporting. |
| Kluska, 2024 (USA)^12^ | Moderate Risk. Although the Renuvion and bipolar RF groups were demographically similar, assignment was based on surgeon preference and clinical need, without randomization or statistical adjustment for confounders such as procedure type, number of concurrent procedures, or patient BMI. | Low Risk. Inclusion was based on consecutive patient treatment records during a defined period, minimizing selection bias. | Moderate Risk. Allocation was not randomized and based on clinical judgment and surgeon preference. This could introduce systematic differences in case complexity or treatment goals. | Moderate Risk. There was likely variability in procedural technique and adjunctive procedures across surgeons and sites, without a standardized protocol. | Low Risk. Adverse event data and procedural metrics were reported for all included patients in both groups; no missing outcome data identified. | Moderate Risk. AE data were extracted from chart review, with no blinding. Subject satisfaction data from the small sub-cohort were subjective and unblinded. No validated outcome scales were used. | Moderate Risk. Although key endpoints (AEs, procedure time) were comprehensively reported, the study lacked predefined primary outcomes and did not include standardized aesthetic or long-term patient-reported measures. | **Moderate Risk.** The study benefits from a large sample size and consistent AE tracking but is limited by its retrospective, non-randomized design, lack of blinding, and absence of standardized efficacy endpoints. These factors contribute to moderate risk across multiple domains despite meaningful comparative insights. |
| Lacerna, 2025 (USA)^13^ | Moderate Risk. The study did not control for baseline differences such as brow asymmetry, age, or skin elasticity, which could influence outcomes. No multivariate analysis or stratification was performed. | Low Risk. All eligible patients treated during the study period were included, reducing selection bias. | Low Risk. The surgical technique was clearly defined and applied uniformly across all participants. | Low Risk. There were no reported deviations from the intended intervention protocol. | Low Risk. Complete follow-up data were reported for all patients, with no loss to follow-up for clinical outcomes. | Low Risk. Aesthetic outcomes were evaluated by independent, blinded reviewers, which minimizes detection bias. Quantitative and patient-reported outcomes were also reported, supporting triangulation of results. | Low Risk. All intended outcomes, including complications and satisfaction metrics, were reported. | **Moderate Risk.** The absence of a control group limits causal inference, but the study's internal consistency, independent blinded outcome evaluation, and thorough reporting reduce the overall risk to a moderate level. |
| Mowlavi, 2020 (USA)^14^ | Moderate risk. No control group was included, and multiple concurrent procedures (UAL + Renuvion) make it difficult to isolate treatment effects. | Low risk. All consecutive patients over a defined time frame were included, minimizing selection bias. | Low risk. Renuvion use is clearly described and uniformly applied across patients. | Low risk. All patients followed the intended protocol. | Low risk. There were no losses to follow-up reported. | Serious risk. Outcomes were based solely on subjective surgeon and patient impressions without standardized tools or blinded assessment, increasing detection bias. | Moderate risk. No objective outcomes or pre-specified endpoints were reported; outcome reporting was limited to general patient satisfaction. | **Serious Risk.**  The study is limited by subjective outcome measures without blinding, absence of a control group, and lack of statistical or objective efficacy endpoints. These factors collectively raise serious concerns about the internal validity of the findings. |
| Nunez Villar, 2024 (Peru)^15^ | Moderate Risk. No adjustment for BMI, age, or prior procedures, though a consistent technique was applied. | Moderate Risk. All patients undergoing the procedure during the specified time period were included; however, non-consecutive enrollment or exclusion criteria were not clarified. | Low Risk. All patients were treated with the same Renuvion-based technique with clear procedural descriptions. | Low Risk. No protocol deviations were reported. | Low Risk. Follow-up data were presented for all patients; no indication of loss to follow-up. | Moderate Risk. Outcomes such as patient satisfaction and fat retention were not objectively measured or validated. | Moderate Risk. Only qualitative findings and select photographic results were reported without statistical analysis. | **Moderate Risk** The absence of objective outcome metrics, control group, and potential for unmeasured confounding factors limit internal validity. However, consistent technique and complete follow-up support a moderate risk rating. |
| Ruff, 2023 (USA)^16^ | Low risk. This was a prospective single-arm IDE study with defined eligibility criteria and standardized treatment, reducing confounding influences. | Low risk. All enrolled patients met predefined inclusion/exclusion criteria under an FDA-reviewed protocol. | Low risk. The study evaluated a single intervention (helium-based plasma RF), and classification was objective and uniform. | Low risk. Treatments were administered per protocol, with no reported deviations. | Low risk. Missing data were minimal. Two patients had missing Day 180 images, which were conservatively handled by using Day 90 images carried forward in the primary endpoint analysis. | Low risk. Independent Physician Reviewers (IPRs) assessed outcomes using blinded, standardized photo comparisons. Patient-reported outcomes were collected via validated instruments. | Low risk. Predefined endpoints were reported in alignment with the protocol and statistical analysis plan. | **Low Risk.** A rigorously conducted, FDA-regulated, single-arm prospective IDE study with structured follow-up, predefined endpoints, and blinded review supports strong internal validity despite the absence of a comparator arm. |
| Ruff, 2020 (USA)^17^ | Serious Risk. No control group was used, and many patients underwent concurrent procedures, including ultrasound-assisted liposuction (UAL). These co-interventions confound the ability to isolate Renuvion’s effects. | Moderate Risk. Inclusion was based on retrospective chart availability and presence of follow-up imaging, which may introduce selection bias and limit generalizability. | Moderate Risk. Device settings and surgical techniques were not standardized across the two chart reviews or clinical sites, which may result in inconsistent exposure to the intervention. | Low Risk. There were no reported deviations from planned interventions in either chart review cohort. | Serious Risk. Significant missing outcome data in Chart Review #1 (only 12 of 37 had complete imaging) and no efficacy assessment in Chart Review #2 (N = 148). This greatly limits the ability to assess treatment effects. | Moderate Risk. Outcomes (aesthetic improvement, satisfaction surveys) were not blinded and relied on subjective, self-reported, and unvalidated assessments, increasing the risk of detection bias. | Low Risk. All prespecified outcomes were reported, including complications, photographic analysis (where available), and patient satisfaction data. | **Serious Risk.** The lack of a control group, presence of confounding interventions, and substantial missing data, particularly for efficacy endpoints, significantly compromise the study's internal validity despite complete AE reporting. |
| Ruff, 2024 (USA)^18^ | Moderate risk. Group allocation was non-randomized and sequential over time, increasing potential for time-based confounding (e.g., evolving surgical technique or patient selection criteria). Baseline characteristics were not fully stratified by group. | Moderate risk. As a retrospective review of consecutively treated patients, selection may have been influenced by clinical factors not equally distributed across groups. No clear matching strategy was described. | Low risk. Group assignment (UAL-only vs. UAL + Renuvion early vs. experienced use) was clearly defined and consistently applied, with no ambiguity in exposure classification. | Low risk. No crossovers or deviations were reported; interventions were delivered as planned. | Low risk. Complete data on adverse events and expected treatment effects were provided for all groups. | Moderate risk. Safety outcomes were clearly defined, but efficacy assessments were anecdotal and not measured using blinded, validated, or quantitative tools, introducing detection bias. | Low risk. All outcomes discussed in the Methods section were reported, including full AE documentation. No evidence of selective omission. | **Moderate Risk.**  This study provides a useful retrospective comparison of safety outcomes across sequential patient groups. However, the lack of randomization, unblinded subjective efficacy assessment, and potential confounding due to time and operator experience limit internal validity. |
| Ruff, 2022 (USA) "Gynecomastia"^19^ | Moderate Risk. Multiple liposuction techniques (UAL, SAL, PAL) were combined with Renuvion without stratified analysis or statistical control for baseline characteristics. This limits causal interpretation of adverse event associations. | Low Risk. All eligible patients during the defined study period were included, reducing risk of selection bias. | Low Risk. Procedural documentation clearly delineated Renuvion-only versus Renuvion + UAL groups. Grouping was consistent and based on operative records. | Low Risk. There were no protocol deviations reported, and all procedures were conducted according to standard practice. | Low Risk. No missing data were reported for adverse event incidence or distribution. | Moderate Risk. Adverse events were not independently adjudicated or blinded, which introduces the potential for reporting or classification bias in a multicenter chart review. | Low Risk. The study comprehensively reported adverse event types, frequencies, and distribution across procedural groups. No evidence of selective reporting. | **Moderate Risk.** This retrospective multicenter study was well-documented and complete for AE reporting, but lack of independent assessment, non-randomized grouping, and heterogeneity in surgical technique reduce its internal validity for comparing safety outcomes. |
| Ruff, 2022 (USA) "Adverse Events"^20^ | Moderate Risk. The study stratified by procedural factors (e.g., liposuction presence, power settings), but patient comorbidities and surgical technique variability across centers may contribute to confounding. | Moderate Risk. Patients were identified from chart reviews without randomization or matching, and inclusion criteria varied by site. | *Low Risk.* Interventions were clearly categorized into Renuvion-only and Renuvion + UAL groups with consistent definitions. | Low Risk. As a retrospective study, deviations were not applicable, and all data were extracted from completed procedures. | Low Risk. The authors reported complete AE data across all participants and sites. | Moderate Risk. Although adverse events were documented using predefined categories, there was no indication that AEs were adjudicated by independent reviewers or blinded assessors. In a multicenter retrospective chart review, subjective variation in how AEs are identified and recorded by site clinicians introduces moderate detection bias. | Low Risk. All planned outcomes related to AEs and seroma risk were reported. | **Moderate Risk.** The study was well-documented and stratified AEs by procedure type. However, the lack of blinding in outcome measurement and center-level variation in AE documentation justifies an overall moderate risk. |
| Shridharani, 2024 (USA)^21^ | Moderate risk. No control group of liposuction-only patients was used; comparisons were made against published literature (Halk meta-analysis). | Moderate risk. Retrospective review; no randomization or consecutive enrollment described. Sites self-selected to contribute data. | Low risk. All patients underwent liposuction followed by helium plasma RF; procedures were clearly defined. | Low risk. No deviations from protocol were described. | Low risk. Safety data was reported for all 483 patients across body areas. | Moderate risk. Adverse events were identified via retrospective chart review, which may lead to underreporting or inconsistent documentation. | Low risk. All adverse events were reported, including by category and body area. | **Moderate Risk.** The study benefits from a large, multicenter sample and low reported AE rates, but the lack of a comparator group, retrospective design, unclear case inclusion strategy, and potential for underreporting or selection bias support a moderate overall risk.. |
| Shridharani, 2022 (USA)^22^ | Moderate Risk. While the absence of a control group and confounder adjustment limits internal validity, the population was relatively uniform (single surgeon, consistent device use). No interventional heterogeneity (e.g., variation in surgical technique or setting) was reported. | Moderate Risk. Patients were included based on availability of records from a single surgeon’s private practice; selection was retrospective. | Low Risk. All patients received liposuction with adjunctive helium plasma RF treatment, and treatment areas were clearly recorded. | Low Risk. All patients received the intended intervention without protocol deviations reported. | Low Risk. Safety data were reported for all 47 patients. No missing outcome data were noted. | Serious Risk. Outcomes were subjectively assessed without standardized tools or blinded evaluation. All safety and efficacy reports relied on chart review and clinician notes. | Moderate Risk. Only descriptive safety outcomes were reported; no pre-specified analysis plan or hypothesis testing was described. | **Moderate Risk.** The study lacks a comparator group, standardized outcome assessments, and blinded evaluation. Outcomes were based on retrospective chart review, limiting internal validity and restricting conclusions to general safety surveillance. |
| Skenderian, 2022 (USA)^23^ | Serious Risk. No control group was included, and results are based solely on subjective surgical outcomes without adjustment for patient-level variables. | Serious Risk. Patients were selected based on subjective assessments of abdominal skin redundancy, with no systematic application of inclusion/exclusion criteria. | Moderate Risk. While the MASR procedure was clearly described, the use of Renuvion was applied at the surgeon’s discretion without standardization. | Moderate Risk. The study does not report deviations from the intended surgical protocol, but adherence is assumed rather than documented. | Serious Risk. No structured safety or efficacy data were presented. Only two complications were described, with no mention of follow-up rates or data completeness. | Serious Risk. Outcomes were not measured using validated scales or objective tools; instead, photographic case examples were used for narrative assessment. | Serious Risk. Results were selectively reported with no predefined outcomes or statistical analysis, increasing the risk of reporting bias. | **Serious Risk.**  The study lacks a control group, includes no quantifiable data, and relies entirely on surgeon-reported outcomes and photographic case illustrations without standardized measurement or systematic follow-up. |
| Sterodimas, 2025 (Greece) “Breast”^24^ | Serious Risk. No control or comparator group was included. Patient factors such as baseline ptosis grade, BMI, age, or skin quality could influence outcomes and were not statistically controlled. | Moderate Risk. Participants were consecutively enrolled, but no eligibility criteria were detailed for standardization beyond presence of mild to moderate ptosis. The study population may not be representative of a broader clinical population. | Low Risk. All patients received the same treatment with the helium-based plasma RF device under standardized procedural parameters, clearly defined in the methods. | Low Risk. There was no evidence that participants deviated from the intended intervention. The procedure was performed consistently. | Low Risk. No missing data were reported; all 15 subjects completed follow-up assessments through Day 180. | Moderate Risk. The primary outcome relied on blinded photographic review, but the authors acknowledged variability in imaging conditions (e.g., lighting, positioning), potentially affecting consistency. No objective imaging tools (e.g., ultrasound, 3D measurement) were used. | Moderate Risk. All planned outcomes appear reported; however, some endpoints (e.g., patient satisfaction) are subjective and potentially influenced by bias, and statistical analysis was limited due to the small sample. | **Serious Risk.** The study lacks a control group and includes a small, non-randomized cohort. Outcomes relied on subjective measures (e.g., GAIS, Breast-Q), and while blinded review was performed, variability in imaging conditions and absence of objective instrumentation (e.g., ultrasound, 3D analysis) may affect reliability. The small sample size and limited statistical analysis further reduce the strength of conclusions, maintaining a serious overall risk of bias despite the prospective design and standardized treatment protocol. |
| Sterodimas, 2025 (Greece) “Lower Eyelid”^25^ | Serious Risk. The study did not control for potential confounders such as baseline skin laxity or age-related differences. | Moderate Risk. Participants were enrolled in a single-arm, prospective clinical study with clear eligibility criteria, but no comparator group. | Low Risk. The intervention was clearly defined and consistently applied across participants. | Low Risk. All procedures were standardized, and no protocol deviations were reported. | Low Risk. Outcome data were available for all participants across all visits. | Serious Risk. Subjective assessments (GAIS, PSQ, and satisfaction surveys) were used without blinding, and photographic review was hampered by quality inconsistency. | Moderate Risk. Results appear to be comprehensively reported, but limitations in photographic quality may influence interpretation. | **Serious Risk.** The lack of a control group, small sample size, and reliance on subjective outcomes present a serious risk of bias, despite prospective design and consistent follow-up. |
| Sterodimas, 2025 (Greece) “Forehead”^26^ | Serious Risk. No comparator group; potential confounders not controlled. | Moderate Risk. Inclusion of patients appears consecutive, but selection criteria not fully described. | Low Risk. Intervention clearly described as helium plasma RF for forehead rejuvenation. | Low Risk. No reported protocol deviations. | Low Risk. All 30 patients completed the satisfaction questionnaire; AE reporting appears complete. | Serious Risk. Use of unblinded self-reported satisfaction surveys and photographic reviewers assessing only 7 patients introduces potential subjectivity and limited generalizability. | Moderate Risk. Results selectively reported; outcomes not clearly pre-specified. | **Serious Risk.** The absence of a control group, retrospective design, and reliance on subjective outcome measures—such as self-reported satisfaction and photographic reviewer assessment in only a subset (7 of 30 patients)—limit the strength of causal inference. Although treatment was standardized and follow-up was complete, the study’s small scale, lack of pre-specified endpoints, and use of unblinded assessments contribute to a serious overall risk of bias. |
| Tambasco, 2024 "Male Chest" (Italy)^27^ | Serious Risk. Patients were not randomized to treatment groups, and allocation depended on clinical presentation (e.g., gland size, ptosis), which introduces selection bias. | Moderate Risk. All patients included had surgical treatment for gynecomastia and were grouped by phenotype, which was clinically appropriate but introduces a risk of overrepresentation in subjective outcome assessments. | Low Risk. Interventions (UAL, Renuvion, skin reduction, fat grafting) were clearly defined per group. | Low Risk. Treatments were delivered according to group designations without reported protocol deviations. | Moderate Risk. Complication and revision rates were reported, but stratified outcome data by group was limited. Aesthetic outcome data were not broken down by severity group. | Serious Risk. Outcomes were subjective and not blinded; aesthetic satisfaction was reported using a non-validated Likert scale, and complications were noted qualitatively. | Moderate Risk. Key results were described, but no statistical analyses or subgroup efficacy comparisons were provided. | **Serious Risk.**  The absence of randomization, reliance on subjective satisfaction metrics, and lack of group-stratified efficacy reporting result in a serious overall risk of bias. |
| Tambasco, 2025 "639 Patients" (Italy)^28^ | Moderate Risk. The study lacked control for key confounding variables such as BMI, age, or baseline skin laxity. While the large sample size adds robustness, the absence of statistical adjustment limits internal validity. | Low Risk. All patients who underwent the combined procedure during the study period were included, reducing the risk of selection bias. | Low Risk. The intervention (UAL + Renuvion) is consistently applied and clearly described. | Low Risk. There’s no indication of deviation from planned procedures. | Low Risk. The study does not report significant missing data; outcome assessments were completed for all patients at the 3-month follow-up. | Low Risk. Although the outcome measures were subjective, they were assessed by two independent, blinded evaluators using predefined scales. A third evaluator resolved any discrepancies, supporting objectivity. | Moderate Risk. The study lacks a pre-registered protocol and statistical analysis plan, raising some concern about selective outcome reporting. | **Moderate Risk.** This large, single-arm retrospective case series was strengthened by the use of blinded evaluators and structured outcome measures. However, the absence of a comparator group, lack of statistical adjustment for confounding variables (e.g., BMI, age, baseline laxity), and no prespecified analysis plan limit the strength of causal inferences, resulting in a moderate overall risk of bias. |
| Tambasco, 2025 “Thighplasty” (Italy)^29^ | Serious Risk. No control group was included for comparison; all patients received the combined procedure. This limits attribution of outcomes specifically to the Renuvion component. | Moderate Risk. Only primary cases were included, and selection bias is possible due to lack of randomization. | Low Risk. Intervention was clearly defined. | Low Risk. The surgical technique appears standardized and performed by a consistent team. | Low Risk. No mention of missing follow-up data; minimum 12-month follow-up for all patients. | Moderate Risk. Outcomes were based on subjective assessment of patient and surgeon satisfaction without validated scales or blinded review. | Moderate Risk. Only favorable results and complication rates are reported; no objective metrics or photographic review is detailed. | **Serious Risk.** The absence of a control group and reliance on subjective assessments without validated tools or blinding limit the strength of the conclusions. Although the intervention was standardized and follow-up complete, the study’s descriptive nature and lack of objective outcome data constrain the reliability of efficacy claims. |
| Tambasco, 2024 "Lipoabdominoplasty" (Italy)^30^ | Moderate Risk. The study does not account for all potential confounding variables such as prior procedures, comorbidities, or surgeon-specific techniques. | Low Risk. Consecutive patients were included, minimizing selection bias. | Low Risk. All patients underwent the same procedure (Renuvion-assisted lipoabdominoplasty), clearly defined. | Low Risk. No crossover or deviation from protocol reported. | Low Risk. No mention of loss to follow-up or missing outcomes. | Serious Risk. Outcomes were based on subjective patient satisfaction ratings without blinding, increasing potential for measurement bias. | Moderate Risk. Limited data provided on complication grading and timing. | **Serious Risk.** The lack of a control group, reliance on subjective outcomes, and absence of blinded assessment increase the risk of bias, despite the well-defined patient cohort and procedure. |
| Troell, 2025 (USA)^31^ | Serious Risk. No control group; outcomes may be influenced by patient selection or concurrent procedures. | Moderate Risk. Consecutive patients were included, reducing but not eliminating selection bias. | Low Risk. All patients underwent a consistent combination of VASER and Renuvion. | Low Risk. No deviations reported. | Low Risk. No significant data omissions were noted. | Serious Risk. Outcomes were subjective (GAIS) and assessed by patients and surgeons, introducing measurement bias. | Moderate Risk. Limited mention of statistical analysis; positive outcomes emphasized. | **Serious Risk.** Despite consistent treatment protocols and complete follow-up, the absence of a comparator group and the use of unblinded, subjective outcome measures (GAIS and surgeon impressions) introduce substantial bias. These limitations reduce confidence in the internal validity of the efficacy conclusions. |
| Vanek, 2024 (USA)^32^ | Moderate Risk. Groups were similar in demographics, but retrospective nature and selection bias possible. | Moderate Risk. Consecutive chart review, but potential for selection bias. | Low Risk. Clear grouping based on use of Renuvion. | Low Risk. No reported deviations. | Moderate Risk. Multivariate analyses excluded up to 30 patients due to missing variables. | Moderate Risk. AE reporting was standardized, but no blinded outcome assessment. | Low Risk. All primary and secondary outcomes reported as planned. | **Moderate Risk.** The retrospective design and incomplete data in multivariate models present limitations, though safety outcomes were systematically assessed across matched groups. |
| Zorrilla, 2022 (USA)^33^ | Serious Risk. The study did not adjust for key variables like baseline pain tolerance, procedure complexity, or perioperative pain management strategies. While it compared groups on some procedural factors (e.g., gluteal fat transfer, BMI), the lack of multivariable adjustment or matching limits causal inference. | Moderate Risk. Although the population included consecutive elective cosmetic patients, the reliance on survey completion introduces risk of nonresponse bias. It’s unclear how many were lost to follow-up or declined participation. | Low Risk. Use of Renuvion was clearly documented and grouped accordingly. The intervention was classified based on surgical records, which is a reliable source. | Low Risk. There is no indication that deviations occurred between intended and delivered interventions. Patients received standard care, and exposure to Renuvion was not influenced by participant behavior. | Moderate Risk. The study does not report the number of patients who were initially enrolled or eligible but did not complete postoperative surveys. Missing outcome data could bias the results, especially if non-responders had different experiences with opioid use or complications. | Moderate Risk. Opioid use was self-reported, introducing recall bias. However, structured postoperative surveys provide some mitigation. | Low Risk. All relevant endpoints were disclosed, including non-significant findings for opioid consumption and complications. | **Moderate Risk.** This rating reflects concerns about confounding, self-reported outcomes, and potentially incomplete follow-up, though the study used consistent exposure classification and reported its results transparently. |

**Supplemental Table 3: Systematic Review Safety & Efficacy Summary Findings**

| **Reference** | **NHMRC Level of Evidence** | **Safety Findings** | **Efficacy Findings** |
| --- | --- | --- | --- |
| Arshad, 2022 (USA)^3^ | IV – Case Series; non-randomized, single-surgeon cohort without a control group. This retrospective study evaluated 20 post-weight-loss patients who underwent Brazilian Butt Lift (BBL) and back contouring procedures with adjunctive use of Renuvion. | The authors reported that the use of helium-based plasma radiofrequency (Renuvion) in back contouring procedures was not associated with any adverse events such as infection, contamination, fat graft loss, or fat embolism. No complications were attributed to Renuvion use. | The authors note that the patients had heightened buttock projection, maximal waistline narrowing, smooth transition line of the waist depicted by the Tilde Curve, generous buttock volume, and resolution of buttock sagging. As well, high patient satisfaction rate. |
| Barone, 2025 (Italy)^1^ | II – Randomized Controlled Trial; prospective, randomized controlled comparison of abdominoplasty with and without Renuvion in post-weight-loss patients (n = 76). | The study reported no significant increase in complications when Renuvion was added:   - Seroma: 5.2% (Renuvion) vs. 2.6% (control) - Hematoma: 0% (Renuvion) vs. 2.6% (control) - Wound dehiscence: 5.2% in both groups - No cases of: skin flap necrosis, burns, or revision surgery   Ultrasound Findings: Faster edema resolution—mean subcutaneous thickness at 6 months was 31 mm (Renuvion) vs. 42 mm (control). | The Renuvion group demonstrated statistically significant improvements across all measured endpoints:   - BODY-Q Satisfaction with Abdomen: Higher at 6 months (p = 0.007), 1 year (p = 0.021), and 2 years (p = 0.024) - BODY-Q Appraisal of Excess Skin: Substantially higher at all time points (p < 0.0001) - VAS Global Aesthetic Ratings: Higher overall (4.9 vs. 4.5, p = 0.01), with improvements in profile, frontal view, and pinch test (all p ≤ 0.01) |
| De La Cruz, 2023 (USA)^4^ | III-2 – Comparative Study with Controls; non-randomized, single-surgeon cohort with concurrent control group. This retrospective review analyzed outcomes in 88 patients who underwent lipoplasty procedures performed by a single surgeon. Patients were divided into two groups: a control group treated with ultrasound-assisted liposuction (UAL-only), and a comparative group (n=25) treated with the SEAL technique, which incorporated adjunctive Renuvion and Microaire power-assisted liposuction with UAL. Data were extracted from historical medical records. | Across the full cohort:   - No thromboembolic events, cardiac complications, or transfusions were reported. - Two seromas occurred in patients treated with Renuvion (2.27%). - One patient (1.13%) developed bullae and subcutaneous emphysema due to helium gas migration following Renuvion application to the left arm.   Other minor complications included (not specified as specific to the Renuvion group):   - Three infections (3.34%) (mild cellulitis resolved with oral antibiotics), - Two cases of brachial plexus neuropraxia from prone positioning (2.27%), - One wound dehiscence following thigh lift (1.13%), - One pressure garment sore (1.13%).   No burns, pneumomediastinum, hospital readmissions, or deaths were reported. No patient required reoperation. | While the primary focus was on safety, the SEAL technique was reported to support favorable aesthetic outcomes in overweight patients. However:   - No quantitative outcomes (e.g., skin laxity reduction, patient satisfaction, or objective contour scoring) were provided. - Efficacy is described qualitatively, with the authors noting that the addition of Renuvion may address residual tissue laxity not corrected by liposuction alone. - No formal comparison between the VASER-only and SEAL groups was made in terms of long-term outcomes or aesthetic effectiveness. |
| DeSouza, 2022 (USA)^5^ | IV – Case Series; retrospective, single-surgeon cohort without control group. This study retrospectively evaluated outcomes in 49 patients undergoing BBL with adjunctive helium-based plasma RF. | No serious adverse events (SAEs) or unanticipated device effects were reported.  Chart review did not identify any adverse events (AEs) attributed to the helium plasma RF device. | Among the 29 patients who completed the Patient Satisfaction Questionnaire (PSQ):   - 86% (25/29) reported being “very satisfied” or “satisfied.” - 93% (27/29) rated their satisfaction 6 or higher on a 10-point scale. - 83% (24/29) would recommend the procedure to others. - 79% (23/29) would consider having the procedure again.   Most patients noted improvements in skin tightness (n=27) and skin feel.  Psychosocial benefits were reported, including improvements in mood (n=24), self-esteem (n=25), and confidence (n=26). |
| Doolabh, 2019 (USA)^6^ | IV – Case Series; non-randomized, single-surgeon cohort without a control group. This retrospective review included 32 patients treated with Renuvion for subdermal coagulation following liposuction. | No intraoperative complications or device-related adverse events were reported. | While the study did not include quantitative skin contraction measures, the author notes favorable short-term clinical outcomes and supports Renuvion's ability to enhance soft tissue contraction. The authors note patients had acceptable final outcomes, with no revisions or secondary procedures required. The study highlights its use across various anatomical areas and describes visible improvements in skin redraping post-liposuction with Renuvion, supported by clinical photographs. |
| Doolabh, 2020 (USA)^7^ | IV – Case Series; non-randomized, single-surgeon cohort without a control group. This retrospective case series describes the outcomes of 15 patients treated by a single surgeon using helium plasma RF technology for subdermal neck coagulation. | The study reported 2 treatment-related adverse events out of 15 patients (13.3%):   - One subject experienced a partial-thickness 0.6 cm³ epidermal lysis in the submental neck crease, which resolved with platelet-rich plasma and amnion treatment. - Another subject experienced transient asymmetry due to likely neuropraxia of the marginal mandibular nerve, which resolved fully after 5 weeks without intervention.   No serious complications such as hematoma, infection, necrosis, or contour irregularities were observed. | Quantitative analysis in 13 patients demonstrated:   - Mean reduction in cervicomental angle: 37.29% (p = .0002) - Mean reduction in submental area: 35.44% (p < .0001) - All patient images (100%) were identified by independent blinded reviewers as showing post-treatment tissue contraction, and 80% of post-treatment images were correctly identified from the image sets. |
| Driscoll, 2024 (USA)^8^ | IV – Case Series; non-randomized, single-center cohort without a control group. This retrospective case series reports on clinical outcomes from 180 patients treated with Renuvion, with or without VASER, across multiple anatomical areas. | The overall complication rate was 13.3%. These included:   - Lymphedema (5%) – all nine cases self-resolved. - Persistent skin laxity (3.3%) – in six patients; two patients required further intervention (one Renuvion retreatment, one excision). - Seromas (2.2%) – 4 cases managed successfully in-office. - Hematomas (1.6%) – 3 cases - Burn at entry point (0.5%) – 1 case due to premature device activation at the incision site. - Neuralgia (0.5%) – 1 case self-limited.   No patient required immediate return to the OR. Risk of complications was associated with elevated helium flow rates (>2 L/min), higher BMI, hypertension, and multiple anatomical sites treated in a single session. | The study did not use validated scales or quantitative aesthetic endpoints. However, authors reported subjective improvements in skin redraping and described treatment-specific Renuvion settings for different anatomical areas. Outcomes were based on clinical observation and surgeon experience. |
| Hoyos, 2025 (Columbia)^9^ | IV – Case Series; non-randomized, single-center cohort without control group. This retrospective series evaluated 174 patients undergoing high-definition liposuction (HDL) with adjunctive Renuvion helium plasma for skin retraction. Ninety-six patients received HDL + Renuvion alone, and 78 had additional ancillary procedures. | The overall complication rate associated with Renuvion-treated body segments was 3.5% (n = 6). No complications required hospitalization or return to the operating room. These included:   - 1 patient (1.04%) developed extended ecchymosis - 1 patient (1.04%) experienced temporary "reedy voice" - 2 patients (2.08%) pneumoperitoneum - 2 patients (2.08%) showed skin overcorrection   Reported in the PSQ (n=101):  48.5% (49/101) report "Severe" Pain  43.6% (44/101) report "Moderate Pain"  7.9% (8/101) report "No Pain" | A patient satisfaction survey (n=100) conducted at 3 months revealed that:   - 44% were ‘very satisfied,’ 41% ‘partially satisfied,’ and 15% ‘unsatisfied.’ - The arms and abdomen were the most favorably rated areas. - 58.6% (58/99) would undergo the procedure again. |
| Ibrahiem, 2022 (Egypt)^10^ | III-2 – Comparative Study with Controls; non-randomized, concurrent groups based on site and patient financial preference. This retrospective study (n=176) compared outcomes across three cohorts: VASER + RFAL, VASER + Renuvion, and VASER alone. Group allocation was not randomized but based on availability and willingness to pay for adjunct technologies. | Out of 66 patients in Group B (VASER + Renuvion):   - Minor burn: 1 case - Seroma: 1 case - Emphysema/crepitus: 3 cases (only reported in Group B) - Asymmetry: 2 cases - Hypertrophic scarring: 2 cases - Revision/Redo procedures: 3 cases   No nerve injuries were observed in Group B (VASER + Renuvion). All complications were managed conservatively without operative intervention. No major complications were reported in any group. Group C (VASER alone) had the highest rate of revision brachioplasty (n = 5). There was no statistically significant difference in complication rates among the three groups (P = .71). | Patient-reported results (VASER + Renuvion):   - Excellent: 27 - Good: 17 - Average: 13 - Poor: 9 - → Total Excellent/Good: 44 of 66 (66.7%)   Independent plastic surgeon review (VASER + Renuvion):   - Excellent: 28 - Good: 21 - Average: 9 - Poor: 8 - → Total Excellent/Good: 49 of 66 (74.2%)   Surgical team review (VASER + Renuvion):   - Excellent: 18 - Good: 34 - Average: 10 - Poor: 4 - → Total Excellent/Good: 52 of 66 (78.8%)   There was no statistically significant difference observed between Group A (RFAL) and Group B (Renuvion). Group C (VASER alone) had consistently poorer aesthetic outcomes and the only instances of revision brachioplasty (n = 5). |
| Khedr, 2024 (Egypt)^11^ | IV – Case Series; non-randomized, single-surgeon cohort without control group. This prospective case series evaluated 46 patients treated with liposuction and adjunctive Renuvion for body contouring. | Complication Rates:   - Postoperative edema: 22 cases (47.8%) - Ecchymosis: 11 cases (23.9%) - Persistent skin laxity (thighs): 1 case (2.2%) - Mild seroma: 3 cases (6.5%) - Pain and tingling: 6 cases (13%) - Mild subcutaneous emphysema: 4 cases (8.7%) - Skin necrosis: 0 cases - Hematoma: 0 cases   No patients required immediate return to the operating room. One patient underwent a follow-up skin-tightening procedure using Renuvion alone for residual laxity. All complications were managed conservatively. | Patient Satisfaction at 6 months:   - Satisfied: 38 patients (82.6%) - Borderline: 7 patients (15.2%) - Unsatisfied: 1 patient (2.2%)   Independent Plastic Surgeon Evaluation (Photographic):   - Excellent: 32 cases (69.6%) - Good: 13 cases (28.3%) - Fair: 1 case (2.1%)   The study notes subjective improvements in skin tone and contour across various treated body areas. However, no objective outcome metrics or validated scoring systems were reported. |
| Kluska, 2024 (USA)^12^ | III-2 – Comparative Study with Controls; non-randomized, single-center cohort with concurrent control group. This retrospective chart review compared outcomes in 229 patients treated with Renuvion versus 236 treated with bipolar RF, with group assignment based on surgeon preference and clinical indication. | In the Renuvion (helium plasma RF) group (N = 229), a total of 45 adverse events were reported across 34 patients. Specific procedure-related events included:   - bruising in 0.4% (1 patient) - burn in 0.4% (1) - edema in 0.9% (2) - epidermolysis in 0.9% (2) - hypertrophic scar in 0.4% (1) - hypesthesia/numbness in 1.3% (3) - anemia in 0.9% (2) - pain in 0.4% (1) - seroma in 4.8% (11) - skin rash in 0.9% (2) - subcutaneous nodule in 0.4% (1) - tissue necrosis in 0.4% (1)   There were no cases of allergic reaction, hematoma, motor nerve weakness, skin hyperpigmentation, or subcutaneous induration reported in the Renuvion group.  In contrast, the InMode bipolar RF group (N = 236) experienced 93 adverse events across 62 patients, reflecting a statistically significantly higher rate of complications compared to Renuvion. The authors specifically noted that fewer occurrences of burns, hematoma, hypertrophic scar, and seroma were reported for helium plasma RF compared with bipolar RF.  Patients in the Renuvion group underwent an average of 4.9 concurrent procedures per case, slightly higher than the 4.4 procedures per case in the bipolar RF group. When evaluating total operative time, including all combined procedures, the Renuvion group demonstrated a shorter average treatment time of 182 minutes compared to 196 minutes in the bipolar RF group. | A subanalysis of nine patients who received both Renuvion and bipolar RF on opposite sides of the body revealed that:   - The Renuvion-treated side required 50% less operative time than the side treated with bipolar RF, suggesting a potential advantage in procedural efficiency. - Overall subject satisfaction was notably higher for areas treated with helium plasma RF at 3- and 6-months posttreatment. - Body area measurement decreased 6.5% at 6 MO in the Renuvion group vs 4.2% in the bipolar group. |
| Lacerna, 2025 (USA)^13^ | IV – Case Series; non-randomized, single-surgeon cohort without control group. This retrospective study evaluated 26 patients who underwent subperiosteal brow lift using helium plasma RF. | Among 26 patients, 3 (11.5%) experienced adverse events: 2 cases of unilateral brow ptosis (1 due to late seroma requiring revision, 1 due to transient CN VII neuropraxia) and 1 case of transient facial nerve palsy. No incidents of scar alopecia, burns, hematoma, infection, or permanent numbness were reported. | Among the 14 patients who consented to photography, 9 had posttreatment images taken ≥90 days after the procedure. In these cases, independent reviewers were able to correctly identify the posttreatment photograph in 78% (7/9) of patients, indicating visible aesthetic improvement. Quantitative measurements performed in 6 patients demonstrated reductions in forehead height ranging from 4.1 to 4.9 mm and increases in brow height ranging from 1.5 to 2.1 mm, supporting both elevation of the brow and shortening of the forehead. Patient-reported outcomes were also favorable: among 15 responses, 87% (n=13) reported being satisfied or very satisfied with the results, and 73% (n=11) indicated they would recommend the procedure to others. |
| Mowlavi, 2020 (USA)^34^ | IV – Case Series; non-randomized, single-surgeon cohort without control group. This single-center, retrospective case series included 14 consecutive patients treated with ultrasound-assisted liposuction (UAL) plus Renuvion. | No complications were reported. The paper states explicitly: “There were no noted complications.” | The paper states: “All patients were satisfied by their aesthetic result and resolution of discomfort associated with preoperative back rolls falling out of their bras.” No objective measurements or validated scales (e.g., GAIS, BODY-Q) were reported for outcome assessment. |
| Nunez Villar, 2024 (Peru)^15^ | IV – Case Series; non-randomized, single-surgeon cohort without control group. This prospective, single-center case series included 220 patients treated by one surgeon using ultrasound-guided helium plasma radiofrequency (Renuvion) for gluteal-hip lipotransfer. | Mild complications were reported in 11 patients (5%). Specifically, temporary hyperthermia was noted in 10 patients (4.5%), all of whom had received more than 800 mL of fat per side; symptoms resolved within 24–48 hours without further intervention. One patient (0.45%) developed retraction of the lower quadrant of the right buttock. Importantly, no major complications were observed. | While no validated quantitative metrics were employed, the authors stated that “the technique allows grafting large volumes of fat and provides greater patient satisfaction compared with previous methods.” Fat grafting volume per buttock ranged from 200 to 1500 mL, with most patients receiving 701–800 mL. Real-time ultrasound guidance confirmed subcutaneous fat placement, and follow-up photos at 4 months in select patients demonstrated maintenance of gluteal contour and volume. The authors emphasized enhanced efficiency and reduced resistance during fat grafting with helium RF pneumodissection, although no direct comparisons or statistical efficacy outcomes were presented. |
| Ouf, 2024 (Egypt)^2^ | II – Randomized Controlled Trial; multiple clinical sites.  This prospective study included 45 individuals with gynecomastia, divided into three groups: Traditional Liposuction, VASER-only, and VASER + Renuvion (n=15 per group). Patients were randomly assigned using a sealed envelope allocation method. Although the sample size was modest, the randomization process qualifies the study as a Level II RCT, supporting an unbiased comparison of safety and efficacy outcomes across groups. | Postoperative Pain: The VASER + Renuvion group had a median pain score of 4 (IQR 3–4), which was significantly lower than the Traditional Liposuction group (5 [IQR 4–6], p = 0.032) and comparable to the VASER-only group (4 [IQR 3–5], p = 0.673). The overall difference was statistically significant (P = 0.023).  Skin Viability: Partial nipple-areola complex (NAC) necrosis was observed in 1 patient (6.7%) in the VASER + Renuvion group — the same rate as the VASER-only group. No necrosis occurred in the Traditional Liposuction group.  Seroma Formation: Reported in 2 patients (13.3%) in the VASER + Renuvion group, compared with 1 patient (6.7%) in each of the Traditional Liposuction and VASER-only groups.  Hematoma: Occurred in 1 patient (6.7%) in the VASER + Renuvion group, 2 patients (13.3%) in the Traditional Liposuction group, and none in the VASER-only group.  Burns: 1 case (6.7%) of skin burn occurred in the VASER + Renuvion group. No burns were reported in the other two groups.  Skin pigmentations were noted in 13.3% of patients in both the Renuvion and VASER-only groups, and 26.7% in the traditional liposuction group; this difference was not statistically significant (P = 0.689).  All complications were considered minor and were resolved conservatively. No major complications or complete NAC loss occurred in any group. | In the VASER + Renuvion group, 93.3% of patients achieved symmetrical breasts, compared to 93.3% in the VASER-only group and 80% in the traditional liposuction group; however, the difference was not statistically significant (P = 0.588).  Regarding volume reduction, 86.7% of patients in the Renuvion group experienced significant reduction, versus 86.7% in the VASER-only group and only 33.3% in the traditional liposuction group. This difference was statistically significant (P = 0.002).  Skin redundancy was reported as minimal in 86.7% of the Renuvion group, compared to 53.3% in the VASER-only group and 40% in the traditional liposuction group. Moderate to significant redundancy was only observed in the traditional and VASER-only groups. The difference in skin redundancy outcomes was statistically significant (P = 0.029).  The need for a second-stage procedure was 0% in the Renuvion group, compared to 13.3% in the VASER-only group and 40% in the traditional liposuction group. This difference was statistically significant (P = 0.017).  Patient satisfaction in the Renuvion group showed that 53.3% were "very satisfied," 40% were "satisfied," and 6.7% were "unsatisfied." In comparison, the VASER-only group had 26.7% very satisfied, 53.3% satisfied, and 20% unsatisfied, while the traditional liposuction group had 0% very satisfied, 60% satisfied, and 40% unsatisfied. The difference in satisfaction levels across groups was statistically significant (P = 0.012).  Objective measurement using the cutometer (R0: UF) values at 6 months showed a mean ± SD of 7.53 ± 2.13 in the Renuvion group, significantly lower (indicating higher firmness) than both the VASER-only group (14.40 ± 3.27) and traditional liposuction group (15.73 ± 2.87). The difference was statistically significant (P < 0.001). |
| Ruff, 2023 (USA)^16^ | IV – Case Series; prospective, single-arm clinical trial without control group. This IDE study prospectively enrolled 65 subjects treated with subdermal helium plasma in the submental area and neck. Although it included structured follow-up and standardized endpoints, the absence of a control group classifies it as a non-comparative case series. | The primary safety endpoint was met, with 96.9% of patients reporting moderate or less pain (defined as a Numerical Rating Scale [NRS] score of 7 or less) during the first 7 days postprocedure (97.5% one-sided lower CL = 89.2%, P < .0001). The highest average pain score reported was 2.3 ± 2.1 on Day 1, which decreased to 0.6 ± 1.2 by Day 7, and all patients returned to baseline pain levels by Day 90.  Expected treatment effects were common and generally mild or self-limiting:   - Edema: 92.3% - Temporary changes in sensory nerve sensation: 86.2% - Bruising: 55.4% - Other effects included erythema, transient crepitus, subcutaneous nodules, and tenderness.   Changes in sensory nerve function were evaluated using the Ten Test, with resolution occurring on average within 76.1 days (10.9 weeks). No interventions were required for resolution of any expected treatment effects.  Adverse events (AEs) were reported as follows:   - Temporary marginal mandibular motor nerve weakness: 6.2% - Pruritus (not associated with nerve changes): 6.2% - Hematoma: 4.6% - Blister formation: 1.5% (due to compression garment) - Hypertrophic scarring: 1.5% (localized to perilobular incisions)   No events of burns or tissue overheating were observed. All AEs resolved without requiring intervention.  Serious adverse events were reported in three patients, including gastrointestinal bleeding, appendicitis, and kidney stone. All were determined to be unrelated to the procedure or the helium plasma device. No device- or procedure-related serious AEs occurred in the study population. | The primary effectiveness endpoint was met, with 82.5% of patients (n = 52) demonstrating improvement in the appearance of lax skin in the neck and submental region at Day 180 based on Independent Physician Review (IPR). This met the prespecified performance goal (97.5% one-sided lower confidence level = 70.9%; P < .0001).  Additional efficacy outcomes at Day 180 included:   - Quantitative lift: Average reduction in surface area of 41 mm², exceeding the success threshold of 20 mm². - Submental volume reduction: Achieved in 68.3% of patients based on Canfield 3D image analysis.   GAIS scores:   - 85.5% of patients rated themselves as “improved” or “much improved.” - 87.1% of investigators reported improved appearance based on physician GAIS.   Patient satisfaction:   - 72.6% of patients reported being happy with their results. - 74.2% would recommend the procedure to a friend. - 75.8% stated they would undergo the procedure on another body area. |
| Ruff, 2020 (USA)^17^  **Note that some data may overlap those from a publication previously summarized herein (Doolabh 2020).* | IV – Case Series; non-randomized, multicenter cohort without control group. This retrospective multicenter case series combined data from two chart reviews (N=37 and N=148) involving patients treated with Renuvion. | In Chart Review #1 (N = 37), a single mild adverse event was reported and determined to be possibly related to the device: localized redness and firmness on the neck, which resolved over 117 days. Three additional events—redness on the buttocks, seroma, and swelling—were considered unrelated and resolved without intervention. No serious or unexpected events were reported in this cohort.  In Chart Review #2 (N = 148), a total of 34 adverse events were documented. The most common event was seroma, occurring in 14 patients (9%), with a mean duration of 44 days. Swelling was reported in three patients (2%) with a mean resolution time of 94 days. Two serious adverse events were recorded: hematomas in the left chest and right chest wall, one required hospitalization and the other aspiration; both subsequently resolved. | In the first chart review cohort (N = 37), quantitative analysis of standardized clinical images demonstrated marked improvements following treatment with helium plasma RF. Mean reductions included 49.2% in neck angle (range, 12.4%–73.6%), 52.0% in submental area (range, 46.1%–67.8%), 26.4% in arm surface area (24.6% on left, 28.1% on right), 82.1% in abdominal width (range, 52%–100%), and 65.3% in abdominal area (range, 29.7%–66.2%)—with the abdominal area data clarified in a subsequent erratum.  Among 37 survey respondents, 75.7% reported improvement in aesthetic appearance, including 40.5% who felt "very much improved," 13.5% "much improved," and 21.6% "improved." Overall satisfaction was high: 74% rated their experience 6–10 on a 10-point scale, and 67.6% indicated they would repeat the procedure or recommend it to others.  Efficacy was not assessed in Chart Review #2. |
| Ruff, 2024 (USA)^18^ | III-2 – Comparative Study with Concurrent Controls; non-randomized, single-center cohort. This single-center study retrospectively evaluated 150 patients divided into three sequential groups: UAL-only (n=50), UAL + Renuvion (early use, n=50), and UAL + Renuvion (experienced use, n=50). | No serious adverse events occurred among the 150 patients. A total of 73 adverse events (AEs) occurred in 48% of patients: 29 in Group 1 (UAL-only), 25 in Group 2 (UAL + Renuvion, early), and 19 in Group 3 (UAL + Renuvion, experienced). The most common AEs were seroma (13%), wound healing issues (10%), and hypertrophic scarring (4%). Expected treatment effects (ETEs) were reported in 89.3% of patients, with edema (74%), pain (69%), and bruising (32%) being most common. Pain peaked in Group 2 (82%) and declined in Group 3 (56%), suggesting a learning-curve effect (P = .013). | While the study primarily focused on safety outcomes and the procedural learning curve, the senior author qualitatively noted an increase in perceived skin contraction when helium plasma was used adjunctively with ultrasound-assisted liposuction (UAL), compared to UAL alone. This perceived improvement was reported to increase over time with greater operator experience. However, no quantitative measures, such as standardized imaging, skin laxity grading, or validated aesthetic scales, were employed to objectively assess efficacy. |
| Ruff, 2022 (USA) "Gynecomastia"^19^ | IV – Case Series; non-randomized, multicenter retrospective cohort without control group. This retrospective chart review included 84 patients across multiple centers who underwent liposuction with adjunctive helium plasma radiofrequency. | No serious adverse events were observed. A total of 5 types of adverse events—swelling, bruising, hematoma, seroma, and diminished SpO₂—were reported in 6 subjects. All events resolved without long-term consequences.  Among device-related events, three seromas were reported: Right hip (mild), right lower quadrant (mild), and right chest (mild). All three cases occurred in areas treated with ultrasound-assisted liposuction (UAL) in conjunction with Renuvion.  Three hematomas were reported: Two in areas treated with UAL + Renuvion: left chest (moderate) and right chest wall (severe). One in an area treated with PAL + Renuvion (mild)  Due to the use of multiple modalities, causality could not be definitively attributed to Renuvion alone. | The study did not assess aesthetic outcomes or patient satisfaction. |
| Ruff, 2022 (USA) "Adverse Events"^20^  **Note that some data may overlap those from a publication previously summarized herein (Ruff 2020).* | III-2 – Comparative Cohort Study; non-randomized, multicenter retrospective analysis with concurrent groups. This retrospective chart review evaluated 192 patients across multiple centers who underwent treatment with Renuvion alone or in combination with ultrasound-assisted liposuction (UAL). | Across the study population, a total of 59 patients experienced 64 adverse events. Detailed Distribution of Adverse Events:   - Seroma: 13 patients experienced a total of 17 seroma events — the most frequent AE reported. - Delayed healing: 3 patients - Swelling: 3 patients - Edema: 2 patients - Epidermal lysis: 2 patients - Fibrosis: 2 patients - Fullness: 2 patients - Other individual AEs (1 patient each): Blepharitis, Blood in urine, Bruising, Conjunctival edema, Drainage, Ectropion, Epiphora, Erythema, Indentation (small), Infected sutures, Inferior dehiscence, Lagophthalmos, Nodule, Open wound (2 events), Photophobia, Capsular contraction, Skin blister, Skin dehiscence, Subcutaneous gas   Seromas were only observed in the Renuvion and UAL group; not in the Renuvion-only group. | This study did not include efficacy endpoints or outcomes related to aesthetic results or patient satisfaction. It focused solely on adverse event reporting and statistical associations with demographic and surgical variables. |
| Shridharani, 2024 (USA)^21^  **Note that some data may overlap those from a publication previously summarized herein (Ruff 2020, Ruff 2022 “Gynecomastia”, Ruff 2022 “Adverse Events”, Shridharani 2022, De Souza).* | IV – Case Series; non-randomized, multicenter retrospective cohort without control group. This retrospective review included 483 patients treated with helium plasma radiofrequency following liposuction across multiple centers. | A total of 32 adverse events (AEs) were reported across 483 patients, representing an overall AE rate of 0.07%. Importantly, no deaths, serious adverse events, embolisms, significant bleeding events, or infections were documented in the entire cohort.  Grouped AE Categories, grouped for direct comparison with published liposuction-only data (e.g., Halk meta-analysis):   - Hematoma/Seroma (4.6%; 22/483): 4 patients experienced hematomas, and 18 patients experienced seromas. - Wound-Related Problems (1.4%; 7/483): Included delayed healing (1), subcutaneous induration (2), subcutaneous nodules (2), and wound complications (2). - Burn/Skin Necrosis/Blister (0.2%; 1/483) - Temporary Nerve Changes (0.4%; 2/483)   The authors analyzed AEs by body area. The results indicated no increased safety risk for any specific body region when using helium plasma RF adjunctively with liposuction compared to liposuction alone. | This analysis did not include any formal efficacy endpoints or measures of aesthetic improvement, patient satisfaction, or skin contraction. |
| Shridharani, 2022 (USA)^22^ | IV – Case Series; non-randomized, single-surgeon cohort without control group. This retrospective study evaluated subdermal helium plasma radiofrequency treatment in 47 patients treated by a single surgeon. | Expected treatment effects were observed in all 47 patients (100%), with the most frequently reported being discomfort, bruising, swelling, and temporary reduction in nerve sensation—each occurring in all patients (24.7% when calculated per ETE event across 190 treated areas). Erythema was reported in only 3 patients (6.4%).  Adverse events were recorded in 22 of the 47 patients (46.8%). The majority (n=20) involved nausea attributed to anesthesia rather than the procedure itself. Isolated events included one case each (2.1%) of transient motor nerve injury and subcutaneous induration. No serious or persistent complications were identified, and all events resolved without intervention. | This retrospective chart review did not include formal quantitative endpoints to evaluate efficacy. However, the authors subjectively noted improvements in skin laxity and contour in all treated areas. No validated scales, blinded assessments, or objective measurements were employed. |
| Skenderian, 2022 (USA)^23^ | IV – Case Series; non-randomized, single-surgeon cohort without control group. This single-center retrospective case series describes 115 Modified Abdominal Skin Resection (MASR) procedures performed by one surgeon. | Safety outcomes were not systematically reported. However, among the 115 MASR cases performed, only two instances of partial skin flap compromise were described, both occurring in patients with undisclosed histories of smoking. These complications resolved with conservative management. The authors emphasize that safety is maintained through limited undermining, preservation of perforators, and avoidance of aggressive liposuction in undermined areas. Renuvion was used as an adjunct when indicated, but no Renuvion-specific adverse events were reported. | Efficacy outcomes are presented anecdotally through case examples rather than quantified clinical endpoints. The authors report that MASR enables maximal excision of redundant skin while preserving critical vascular structures, facilitating simultaneous high-definition ultrasound-assisted liposuction. According to the authors’ experience with 115 cases, this approach yields superior aesthetic outcomes including waistline narrowing, enhanced muscle highlights, and smoother contour transitions. Renuvion was selectively used in cases with moderate upper abdominal skin laxity to optimize contraction. No validated patient-reported outcome measures or objective skin contraction metrics were reported. |
| Sterodimas, 2025 (Greece) “Breast”^24^ | IV – Case Series; non-randomized, single-surgeon cohort without control group. This prospective study followed 15 patients treated with subdermal helium plasma radiofrequency in the breast by a single surgeon. | No adverse events (AEs) or expected treatment effects (ETEs) were reported across any time points.   - Pain: The average pain score was zero throughout the study. - Intraoperative and Postoperative Tolerance: Patients reported no pain during the procedure or at any follow-up visits. - Complications: No instances of bruising, swelling, sensory changes, burns, infections, seromas, or other AEs were observed. | Photographic Assessment (IPR): Improvement was observed in 67% of patients at Day 90 and 73% at Day 180.  Morphometric Analysis: Vertical lifting was demonstrated by reductions in SSN–nipple distance (−1.2 cm), SSN–base (−1.5 cm), and other key breast measurements, indicating soft tissue contraction and glandular repositioning.  Ptosis Grade Improvement: By Day 180, 40% of patients improved from Grade II to Grade I ptosis. The remainder remained at Grade II.  Patient & Investigator GAIS Ratings: At Day 180, 100% of patients were rated as improved—67% as “very much improved” and 33% as “much improved”—by both patients and investigators.  Patient-Reported Outcomes: Breast-Q scores improved across all domains, particularly body confidence and self-perception. All patients reported less sagging, improved nipple position, and a more youthful appearance, with 100% willing to recommend the procedure. |
| Sterodimas, 2025 (Greece) “Lower Eyelid”^25^ | IV – Case Series; non-randomized, single-surgeon cohort without control group. This prospective study followed 16 patients treated with subdermal helium plasma radiofrequency in the lower eyelid by a single surgeon. | The average pain score reported across all time points was zero, with no intraoperative or postoperative pain reported.  The most common expected treatment effects (ETEs) were bruising (87.5%) and edema (81.3%), which are anticipated side effects in procedures involving tissue undermining.  One adverse event was reported, contact dermatitis, which was mild, resolved without intervention, and not serious. No serious adverse events (SAEs) or cases of postoperative chemosis were reported. | 15 subjects completing D90 and D180.  Photographic Review (IPR): Success rates were 53% at Day 90 and 33% at Day 180. Low rates were attributed to inconsistencies in pre/posttreatment image quality.  Objective Measures: All subjects improved to grade 0 (normal) on the snap-back test by Day 180. Regarding Canthal Laxity Test Results, at baseline, nearly all subjects demonstrated medial and lateral canthal laxity, primarily at grade I. Following treatment, marked improvement was observed. By day 30, 86.7% of subjects had improved to grade 0 for both medial and lateral canthal laxity, with the remainder at grade I. By days 90 and 180, 100% of subjects had achieved grade 0 laxity in both medial and lateral canthal regions.  Global Aesthetic Improvement: Investigators rated 93.3% of patients as “very much improved” and 6.7% as “much improved” at Day 180. Patients self-rated similarly: 66.7% “very much improved” and 33.3% “much improved.”  Patient Satisfaction: All patients (100%) reported satisfaction and said they would recommend the procedure. Most common reported improvements included reduced sagging (80%) and smoother skin texture (73.3%). |
| Sterodimas, 2025 (Greece) “Forehead”^26^ | IV – Case Series; non-randomized, single-surgeon cohort without control group. This retrospective review analyzed outcomes in 30 patients treated with Renuvion for forehead rejuvenation by a single surgeon. | - No serious adverse events were reported. - A total of 11 expected treatment effects occurred in 7 patients (23.3%): Bruising in 6 patients (20%) and Edema/swelling in 5 patients (16.7%) - All events resolved without intervention, most by Day 10.   No unanticipated or persistent complications were observed. | Photographic Reviewer Assessment: Independent reviewers correctly identified the post-treatment photo in 71% (5/7) of cases.  Patient Satisfaction Questionnaire (PSQ):   - 100% (30/30) reported visible improvement in the treated area. - 100% said they would recommend the procedure. - Most frequently reported improvements included: Reduction in forehead lines (56.7%), Eyebrow elevation (46.7%), More youthful appearance (46.7%) - All patients reported satisfaction: 63.3% (19/30) “very satisfied”, 36.7% (11/30) “satisfied”. |
| Tambasco, 2024 "Male Chest" (Italy)^27^ | IV – Case Series; non-randomized, single-surgeon cohort without control group. This retrospective chart review included 300 male patients treated for gynecomastia across phenotype-based grades I–IV. Outcomes were reported following a multimodal surgical approach incorporating ultrasound-assisted liposuction (UAL) with or without Renuvion, fat grafting, and skin reduction techniques. Renuvion was utilized in 187 patients. | 226 patients were included in Grades II-IV where Renuvion could have been utilized. The following AEs were reported for those grades:   - Seroma: 4 Grade II cases, 2 Grade III cases, 3 Grade IV cases - Hematoma: 1 Grade III case, 1 Grade IV case - Infection: 1 Grade IV case - Hyperpigmentation: 2 Grade II cases, 3 Grade III cases - Vascular compromise: 1 Grade IV case - Pulmonary embolism: 1 Grade IV case - Serious infection requiring IV antibiotics: 1 Grade IV case - Wound dehiscence requiring surgery: 2 Grade IV cases. - Revision surgery: 11 required further correction through a secondary vertical approach (Grades unknown), 5 for unpleasant scars with 3 Grade III and 2 Grade IV | Patient Satisfaction: 100% of patients rated results as either “satisfactory” (12%) or “very satisfactory” (88%) on a 5-point Likert scale. |
| Tambasco, 2025 "639 Patients" (Italy)^28^ | IV – Case Series; non-randomized, single-arm retrospective study without a control group. This retrospective analysis of 639 patients evaluated outcomes following combined UAL and helium plasma RF treatment. | Among 639 patients treated with combined ultrasound-assisted liposuction (UAL) and Renuvion, the overall complication rate was low. Minor complications included subcutaneous emphysema (2.3%), hyperpigmentation (1.25%), seroma (5.6%), and hematoma (1.1%). Major complications were rare, with no reported cases of burns or venous thromboembolism, and a low infection rate of 0.3%. | 87% of patients were judged to have no residual skin laxity, and 91% were reported to have achieved excellent body contouring improvement. The remaining patients showed moderate laxity (13%) or good contour improvement (9%). These evaluations were based on baseline and 3-month follow-up images reviewed by two independent blinded assessors using separate 3-point scales for skin laxity and contour improvement. In cases of disagreement, a third blinded evaluator provided the final assessment. |
| Tambasco, 2025 “Thighplasty” (Italy)^29^ | IV – Case Series; non-randomized, single-center cohort without control group. This descriptive case series (n = 21) reports outcomes from a single-center experience treating patients with combined J-pattern medial thighplasty and Renuvion-assisted liposuction. | The study reports the absence of major complications. The following minor complications were reported:   - 2% (1/21) Seroma - 2% (1/21) Hyperpigmentation - 2% (1/21) Minor wound dehiscence | The authors state that the combination approach was effective and reproducible in treating medial thigh laxity. Reported efficacy outcomes included:   - Improved aesthetic outcomes attributed to skin tightening and enhanced skin tone from HPFR. - Subjective claims of high patient satisfaction, linked to shorter, well-concealed scars and favorable postoperative contour. - HPFR was credited with immediate and progressive tissue contraction, stimulating collagen formation and reducing scar tension. - While no quantitative patient-reported outcomes (e.g., satisfaction scores or GAIS) or photographic review results were presented, the authors conclude that the combined procedure yielded positive results in a post-bariatric population. |
| Tambasco, 2024 "Lipoabdominoplasty" (Italy)^30^ | IV – Case Series; non-randomized, single-surgeon cohort without control group. This retrospective review analyzed outcomes in 100 consecutive patients who underwent Renuvion-assisted lipoabdominoplasty. | Minor complications included:   - Hyperpigmentation: 8% - Seroma: 6% - Subcutaneous emphysema: 2% - Hematoma: 2% - Wound dehiscence: 1%   No cases of infection, burns, or venous thromboembolism were reported.  No patient required surgical revision due to complications. | All patients reported satisfactory outcomes. 88% rated their result as 5/5 on a Likert scale; the remaining 12% rated 4/5.  No patients required revision surgery to improve the cosmetic result.  While formal objective measures were not presented, the high satisfaction rates suggest a positive aesthetic effect from the procedure. |
| Troell, 2025 (USA)^31^ | IV – Case Series; non-randomized, multi-surgeon cohort without control group. This retrospective study included 58 patients treated with ultrasound-assisted liposuction (VASER) combined with helium-based plasma RF (Renuvion) for lower face and/or neck contouring between 2018 and 2022 across two private practices. | - No serious adverse events occurred. - Temporary marginal mandibular nerve weakness: 12% (7/58), all resolved within 1–6 weeks. - Minor contour irregularities: 15.5% (9/58); 2 patients required dermal filler for persistent jowl depressions. - Revision procedures: 6.9% (4/58), including liposuction touch-ups (morbidly obese). - Expected side effects: Mild discomfort, ecchymosis, edema, and itching; specific percentages not reported. - No reports of helium embolism, burns, infection, hematoma, seroma, scarring, or pigment changes. | Patient Satisfaction (GAIS):   - 95.5% (56/58) reported improvement. - 2 patients (3.4%) reported “no change.” - No “poor” aesthetic outcomes were recorded.   Surgeon Assessment:   - Observed superior skin retraction and skin tightening with the addition of helium plasma RF compared to ultrasound alone. - Improvement noted as early as 24 hours post-procedure, with progressive results over 6 months. |
| Vanek, 2024 (USA)^32^ | III-2 – Comparative Study with Controls; non-randomized, retrospective, single-center cohort study evaluated 77 patients who underwent lipoabdominoplasty, comparing those treated with ultrasound-assisted liposuction (UAL) alone versus UAL with adjunctive Renuvion. Safety outcomes were assessed through clinical chart review and multivariate regression analysis, supporting comparative inference despite the absence of randomization. | Adverse Events requiring Hospitalization, Home Health or Emergency Room Visit: Occurred in 3 of 40 patients (7.5%) in the Renuvion group and 3 of 37 patients (8.1%) in the control group. Renuvion group SAEs included abdominal wall cellulitis (n=1), tissue necrosis (n=1), and wound dehiscence (n=1). No statistically significant difference was found between groups (P = .628).  Renuvion group in-office interventions for adverse events: seromas (n=6), tissue necrosis (n=1), and wound dehiscence (n=4).  Nonsignificant adverse events for Renuvion Group: delayed healing (n=3) and 1 each for fibrosis, superficial thrombophlebitis, and wound dehiscence. All nonsignificant AEs resolved without long-term sequelae.  Multivariate analysis: No variable, including treatment with helium plasma RF, age, BMI, weight loss history, smoking status, or extent of skin excision, was found to be a statistically significant predictor of adverse events. Three multivariate logistic regression models confirmed these findings. | This study did not include direct measures of efficacy such as aesthetic outcomes or patient satisfaction. |
| Zorrilla, 2022 (USA)^33^ | III-2 – Comparative Study with Controls; non-randomized, prospective observational study included 302 patients undergoing elective outpatient cosmetic procedures, with 21 patients receiving helium plasma RF as part of their treatment. Outcomes, including opioid consumption, were evaluated across groups, enabling comparison between those who received Renuvion and those who did not. | The authors reported low complication rates overall. Specifically, among the Renuvion cohort, two patients (9.5%) experienced minor complications: one wound infection and one seroma. Both were managed conservatively in the outpatient setting without the need for hospitalization or surgical revision. No serious adverse events were reported in this group.  The primary endpoint of the study was postoperative opioid consumption, used as a proxy for pain and recovery burden. Across all cosmetic surgery types, patients consumed an average of 13.8 opioid pills, despite being prescribed an average of 28. In the subset of procedures involving helium plasma RF, the mean number of opioid pills consumed was slightly higher at 14.3, though this difference was not statistically significant (P = .8422). Additionally, the study found no significant difference in opioid use between patients who underwent liposuction with gluteal fat transfer with or without helium plasma RF. These results suggest that the addition of helium plasma RF did not increase postoperative discomfort requiring opioid analgesia, supporting its role as a well-tolerated adjunctive technology in body contouring procedures. | This study did not include direct measures of efficacy such as aesthetic outcomes or patient satisfaction. |

Supplemental Table 4: Characteristics of included studies

| **Study Characteristics** | | | | | | **Participant Characteristics** | | | **Exposure/Intervention** | | | **Outcomes** |
| --- | --- | --- | --- | --- | --- | --- | --- | --- | --- | --- | --- | --- |
| **First Author, Year** | **Country** | **Design** | **Procedure Type** | **Study Sponsor** | **Average Follow-up (months)** | **Sample Size** | **Average Age** | **Sex (%F / %M)** | **Treated Areas** | **Concomitant Treatments** | **Treatment** | **Categories** |
| Arshad, 2022 | USA | Case Series | Combination | Independent/IIS | . | 20 | . | NM | Back; Leg | Ultrasonic-assisted liposuction | Power: 80; LPM: 3; Passes: 6 | Safety; Other efficacy |
| Barone, 2025 | Italy | Randomized Controlled Trial | Combination | Independent/IIS | 24 | 38 | 34.2 | 61% / 39% | Abdomen/pubis; Hip/Flank | UNK liposuction; Excess skin resection | Power: 70-80; LPM: 2.5; Passes: 6 | Safety; Other efficacy |
| De La Cruz, 2023 | USA | Comparative Study with Controls | Renuvion + Lipo | Independent/IIS | 1 | 25 | 40.8 | 72% / 28% | Abdomen/pubis; Arms; Back; Hip/Flank; Leg | Ultrasonic-assisted liposuction; MicroAire liposuction; Excess skin resection | Power: 80; LPM: 2; Passes: 5 | Safety |
| DeSouza, 2022 | USA | Case Series | Renuvion-only | Sponsored | . | 49 | 32 | 100% / 0% | Abdomen/pubis; Arms; Back; Hip/Flank | UNK liposuction | Power: 80; LPM: 2 - 2.5; kJ: 6 | Satisfaction; Safety; Other efficacy |
| Doolabh, 2019 | USA | Case Series | Renuvion + Lipo | Sponsored | 6 | 32 | 48 | 91% / 9% | Abdomen/pubis; Arms; Back; Buttocks; Chest/axilla; Hip/Flank; Leg; Neck | Ultrasonic-assisted liposuction | Power: 61.9 (50 - 80); LPM: 3.25 (2 - 4); Passes: 6.2 (6 - 8) | Safety; Other efficacy |
| Doolabh, 2020 | USA | Case Series | Renuvion-only | Sponsored | 4 | 15 | 62 | 93% / 7% | Neck | None | Power: 70 (60-80); LPM: 2 (1.5-4); Passes: 5 (3-6) | IPR; Safety |
| Driscoll, 2024 | USA | Case Series | Renuvion + Lipo | Independent/IIS | . | 180 | 45.4 | 89% / 11% | Abdomen/pubis; Arms; Back; Face/submental; Hip/Flank; Leg | Ultrasonic-assisted liposuction | Power: Abd 80 (80 - 90) Arms 80 (70 - 80) Back 90 (80 - 90) Hip 90 (80 - 90) Leg 80 (80 - 80) Face/Sub 70 (70 - 70); LPM: Abd 2 (2 - 2) Arms 1.5 (1.5 - 2) Back 2 (2 - 2) Hip 2 (2 - 2) Leg 2 (1.5 - 2) Face/Sub 1.5 (1 - 1.5); kJ: Abd 10.5 (6.5 - 14.5) Arms 10 (7.05 - 14) Back 10 (8 - 15) Hip 10 (8 - 12.9) Leg 10 (6 - 11) Face/Sub 3 (2 - 4) | Safety; Other efficacy |
| Hoyos, 2025 | Columbia | Case Series | Renuvion + Lipo | Independent/IIS | 7.5 | 96 | 39.4 | 85% / 15% | Abdomen/pubis; Arms; Back; Buttocks; Chest/axilla; Hip/Flank; Leg; Neck | Ultrasonic-assisted liposuction; MicroAire liposuction | NM | Satisfaction; Safety |
| Ibrahiem, 2022 | Egypt | Comparative Study with Controls | Renuvion + Lipo | Independent/IIS | 13 | 66 | 32 | 86% / 14% | Arms; Chest/axilla | Ultrasonic-assisted liposuction | Power: 60; LPM: 1.5; Passes: 6 | Inv GAIS; Pt GAIS; IPR; Safety |
| Khedr, 2024 | Egypt | Case Series | Renuvion + Lipo | Independent/IIS | 6 | 46 | 40.93 | 87% / 13% | Abdomen/pubis; Arms; Back; Leg; Neck |  | kJ: 7 + 1.3 (4.4-10.5) | Satisfaction; IPR; Safety |
| Kluska, 2024 | USA | Comparative Study with Controls | Renuvion + Lipo | Sponsored | 10.3 | 229 | 42.4 | 89% / 11% | Abdomen/pubis; Arms; Back; Chest/axilla; Face/submental; Hip/Flank; Leg; Neck | Ultrasonic-assisted liposuction; Suction-assisted liposuction | NM | Safety; Other efficacy |
| Lacerna, 2025 | USA | Case Series | Renuvion-only | Sponsored | 9.8 | 26 | 61 | 96% / 4% | Face/submental | None | Power: 75 (30-80) Micro 30; LPM: 1.6 (1.2-2) Micro 1; kJ: 4.8 (1.5-6) Micro 6 | Satisfaction; Safety; Other efficacy |
| Mowlavi, 2020 | USA | Case Series | Renuvion + Lipo | Independent/IIS | 6 | 14 | 39 | 100% / 0% | Back | Ultrasonic-assisted liposuction | Power: 80; LPM: 3; Passes: 6 | Safety; Other efficacy |
| Nunez, 2024 | Peru | Case Series | Renuvion + Lipo | Independent/IIS | 12 | 220 | 48.5 | 96% / 4% | Buttocks | Fat transfer | Power: 70; LPM: 2.5; Passes: 1-2 | Safety |
| Ouf, 2024 | Egypt | Randomized Controlled Trial | Renuvion + Lipo | Independent/IIS | 6 | 15 | 31.5 | 0% / 100% | Chest/axilla | Ultrasonic-assisted liposuction | Power: 70; LPM: 3 | Satisfaction; Safety; Other efficacy |
| Ruff, 2020 CR1 | USA | Case Series | Renuvion + Lipo | Sponsored | 3.5 | 37 | 45.5 | 84% / 16% | Abdomen/pubis; Arms; Back; Buttocks; Chest/axilla; Face/submental; Hip/Flank; Leg; Neck | Ultrasonic-assisted liposuction; Suction-assisted liposuction; UNK liposuction | Power: 70 (40 - 85); LPM: 3 (1.5 - 4); Passes: 6 (1.5 - 6) | Pt GAIS; Satisfaction; IPR; Safety; Other efficacy |
| Ruff, 2020 CR2 | USA | Case Series | Renuvion + Lipo | Sponsored | . | 148 | 52.5 | 81% / 19% | Abdomen/pubis; Arms; Back; Buttocks; Chest/axilla; Face/submental; Hip/Flank; Leg; Neck | Ultrasonic-assisted liposuction; Laser-assisted liposuction; UNK liposuction | Power: 70 (20 - 100); LPM: 3 (1.5 - 4); Passes: 4 (2 - 9) | Safety |
| Ruff, 2022 AE | USA | Comparative Study with Controls | Combination | Sponsored | 3 | 192 | 47.8 | 85% / 15% | Abdomen/pubis; Arms; Back; Buttocks; Chest/axilla; Face/submental; Hip/Flank; Leg; Neck | Ultrasonic-assisted liposuction | Power: (40 - 100); LPM: (1.5 - 4); Passes: 2 - 11 | Safety |
| Ruff, 2022 Gyn | USA | Case Series | Combination | Sponsored | 1.5 | 84 | 40 | 0% / 100% | Chest/axilla | Ultrasonic-assisted liposuction; Suction-assisted liposuction; Power-assisted liposuction | Power: 75; LPM: 3; Passes: 5 | Safety |
| Ruff, 2023 | USA | Case Series | Renuvion-only | Sponsored | 6 | 65 | 55.9 | 91% / 9% | Face/submental | None | Power: 70; LPM: 1.5; Passes: 4 - 6 | Inv GAIS; Pt GAIS; Satisfaction; IPR; Safety; Other efficacy |
| Ruff, 2024 | USA | Case Series | Combination | Sponsored | 10.2 | 100 | 46.8 | 76% / 24% | Abdomen/pubis; Arms; Back; Buttocks; Chest/axilla; Face/submental; Hip/Flank; Leg; Neck | Ultrasonic-assisted liposuction | NM | Safety |
| Shridharani, 2022 | USA | Case Series | Renuvion-only | Independent/IIS | 6 | 47 | 45 | 83% / 17% | Abdomen/pubis; Arms; Back; Buttocks; Chest/axilla; Hip/Flank; Leg; Neck | Suction-assisted liposuction | Power: 70 (60 - 80); LPM: 1.5 (1.5 - 2); Passes: 4 - 6 | Safety |
| Shridharani, 2023 | USA | Case Series | Renuvion + Lipo | Sponsored | . | 483 | 44.7 | 71% / 29% | Abdomen/pubis; Arms; Back; Buttocks; Chest/axilla; Face/submental; Hip/Flank; Leg; Neck | UNK liposuction | NM | Safety |
| Skenderian, 2022 | USA | Case Series | Combination | Independent/IIS | . | 115 | . | NM | Abdomen/pubis | Ultrasonic-assisted liposuction; Excess skin resection | Power: 80; LPM: 3; Passes: 6 | Safety; Other efficacy |
| Sterodimas, 2025 Br | Greece | Case Series | Renuvion-only | Sponsored | 6 | 15 | 38 | 100% / 0% | Chest/axilla | None | Power: 70; LPM: 1.5; Passes: 6; kJ: 14.6 + 3.7 | Inv GAIS; Pt GAIS; Satisfaction; IPR; Safety; Other efficacy |
| Sterodimas, 2025 Fo | Greece | Case Series | Renuvion-only | Sponsored | 27 | 30 | 54 | 83% / 17% | Face/submental | None | Power: 65 (60 - 75); LPM: 1; Passes: 4; kJ: 2.4 (1.6 - 2.8) | Pt GAIS; Satisfaction; Safety; Other efficacy |
| Sterodimas, 2025 LoEy | Greece | Case Series | Renuvion-only | Sponsored | 6 | 16 | 48.1 | 81% / 19% | Face/submental | None | Power: 60 - 65; LPM: 1; Passes: 3; kJ: 0.8 + 0.3 | Inv GAIS; Pt GAIS; Satisfaction; IPR; Safety; Other efficacy |
| Tambasco, 2024 639 | Italy | Case Series | Renuvion + Lipo | Independent/IIS | 25 | 639 | 31.5 | 78% / 22% | Abdomen/pubis; Arms; Back; Hip/Flank; Leg; Neck | Ultrasonic-assisted liposuction; Excess skin resection | Power: 80; LPM: 1.5 - 3.5; Passes: 3 - 6 | IPR; Safety |
| Tambasco, 2024 Lipoab | Italy | Case Series | Combination | Independent/IIS | 19.7 | 100 | 37 | 83% / 17% | Abdomen/pubis | Ultrasonic-assisted liposuction; Excess skin resection | Power: 80; LPM: 2.5 | Satisfaction; Safety |
| Tambasco, 2024 MaCh | Italy | Case Series | Combination | Independent/IIS | 16.8 | 226 | 34.8 | 0% / 100% | Chest/axilla | Ultrasonic-assisted liposuction | NM | Pt GAIS; Safety |
| Tambasco, 2025 | Italy | Case Series | Combination | Independent/IIS | . | 21 | 30.4 | NM | Leg | Traditional liposuction; Excess skin resection | Power: 80; LPM: 2.5; Passes: 3 - 6 | Safety; Other efficacy |
| Troell, 2025 | USA | Case Series | Renuvion + Lipo | Independent/IIS | 6 | 58 | 36 | NM | Face/submental; Neck | Ultrasonic-assisted liposuction | Power: 70 - 80; LPM: 1.5 - 3.0; Passes: 6 | Pt GAIS; Safety |
| Vanek, 2025 | USA | Comparative Study with Controls | Combination | Sponsored | . | 40 | 53.2 | 93% / 8% | Abdomen/pubis | Ultrasonic-assisted liposuction; Excess skin resection | Power: 80; LPM: 2.4; Passes: 6 | Safety |
| Zorilla, 2022 | USA | Comparative Study with Controls | Combination | Independent/IIS | . | 21 | 35.35 | NM | Unknown |  | NM | Safety |

*Definitions: Not Mentioned (NM)*

**Supporting Statistical Results**

Additional statistical analyses include meta regression results, funnel plots, sensitivity analyses, and average follow-up analyses that were conducted but not included in the main body of the manuscript.

**Meta regression results for all endpoints using follow-up (days) as a predictor:**

| **Endpoint** | **N studies** | **I^2^ (%)** | **Beta coefficient** | **95% Confidence Interval** | **p-value** |
| --- | --- | --- | --- | --- | --- |
| **Efficacy** |  |  |  |  |  |
| Patient Satisfaction | 10 | 66.31 | 0.037 | (0.012, 0.061) | 0.003 |
| Investigator GAIS | 4 | 59.47 | -0.070 | (-0.165, 0.024) | 0.144 |
| Patient GAIS | 8 | 89.99 | 0.021 | (-0.021, 0.063) | 0.325 |
| IPR | 8 | 90.12 | 0.035 | (-0.021, -0.090) | 0.220 |
| **Safety** |  |  |  |  |  |
| Overall Complications | 25 | 87.47 | -0.005 | (-0.025, 0.014) | 0.818 |

**A: Patient Satisfaction Funnel Plot**

**B: Investigator GAIS Funnel Plot**

**C: Patient GAIS Funnel Plot**

**D: IPR Funnel Plot**

**E. Complications Funnel Plot**

**A. Sensitivity analysis, leave one out – Patient Satisfaction**

**B. Sensitivity analysis, leave one out – Investigator GAIS**

**C. Sensitivity analysis, leave one out – Patient GAIS**

**D. Sensitivity analysis, leave one out - IPR**

**E. Sensitivity analysis, leave one out - Complications**

**Supplemental Table 5: PRISMA 2020 Checklist**

| **Section and Topic** | **Item #** | **Checklist item** | **Location where item is reported** |
| --- | --- | --- | --- |
| **TITLE** | | |  |
| Title | 1 | Identify the report as a systematic review. | Title page |
| **ABSTRACT** | | |  |
| Abstract | 2 | See the PRISMA 2020 for Abstracts checklist. | Abstract |
| **INTRODUCTION** | | |  |
| Rationale | 3 | Describe the rationale for the review in the context of existing knowledge. | Introduction |
| Objectives | 4 | Provide an explicit statement of the objective(s) or question(s) the review addresses. | Introduction |
| **METHODS** | | |  |
| Eligibility criteria | 5 | Specify the inclusion and exclusion criteria for the review and how studies were grouped for the syntheses. | Methods – Inclusion/Exclusion Criteria |
| Information sources | 6 | Specify all databases, registers, websites, organisations, reference lists and other sources searched or consulted to identify studies. Specify the date when each source was last searched or consulted. | Methods – Data Sources and Search Strategy |
| Search strategy | 7 | Present the full search strategies for all databases, registers and websites, including any filters and limits used. | Supplemental Materials (Search Strategy) |
| Selection process | 8 | Specify the methods used to decide whether a study met the inclusion criteria of the review, including how many reviewers screened each record and each report retrieved, whether they worked independently, and if applicable, details of automation tools used in the process. | Methods – Data Sources and Search Strategy |
| Data collection process | 9 | Specify the methods used to collect data from reports, including how many reviewers collected data from each report, whether they worked independently, any processes for obtaining or confirming data from study investigators, and if applicable, details of automation tools used in the process. | Methods – Data Extraction and Quality Assessment |
| Data items | 10a | List and define all outcomes for which data were sought. Specify whether all results that were compatible with each outcome domain in each study were sought (e.g. for all measures, time points, analyses), and if not, the methods used to decide which results to collect. | Methods – Data Extraction and Quality Assessment |
|  | 10b | List and define all other variables for which data were sought (e.g. participant and intervention characteristics, funding sources). Describe any assumptions made about any missing or unclear information. | Methods – Data Extraction and Quality Assessment |
| Study risk of bias assessment | 11 | Specify the methods used to assess risk of bias in the included studies, including details of the tool(s) used, how many reviewers assessed each study and whether they worked independently, and if applicable, details of automation tools used in the process. | Methods – Data Extraction and Quality Assessment |
| Effect measures | 12 | Specify for each outcome the effect measure(s) (e.g. risk ratio, mean difference) used in the synthesis or presentation of results. | Methods – Meta-Analysis |
| Synthesis methods | 13a | Describe the processes used to decide which studies were eligible for each synthesis (e.g. tabulating the study intervention characteristics and comparing against the planned groups for each synthesis (item #5)). | Methods – Meta-Analysis |
|  | 13b | Describe any methods required to prepare the data for presentation or synthesis, such as handling of missing summary statistics, or data conversions. | Methods – Meta-Analysis |
|  | 13c | Describe any methods used to tabulate or visually display results of individual studies and syntheses. | Methods – Meta-Analysis |
|  | 13d | Describe any methods used to synthesize results and provide a rationale for the choice(s). If meta-analysis was performed, describe the model(s), method(s) to identify the presence and extent of statistical heterogeneity, and software package(s) used. | Methods – Meta-Analysis |
|  | 13e | Describe any methods used to explore possible causes of heterogeneity among study results (e.g. subgroup analysis, meta-regression). | Methods – Meta-Analysis |
|  | 13f | Describe any sensitivity analyses conducted to assess robustness of the synthesized results. | Methods – Meta-Analysis |
| Reporting bias assessment | 14 | Describe any methods used to assess risk of bias due to missing results in a synthesis (arising from reporting biases). | Methods – Meta-Analysis |
| Certainty assessment | 15 | Describe any methods used to assess certainty (or confidence) in the body of evidence for an outcome. | Not performed |
| **RESULTS** | | |  |
| Study selection | 16a | Describe the results of the search and selection process, from the number of records identified in the search to the number of studies included in the review, ideally using a flow diagram. | Results – Study Selection and Characteristics |
|  | 16b | Cite studies that might appear to meet the inclusion criteria, but which were excluded, and explain why they were excluded. | PRISMA Figure (Figure 1) |
| Study characteristics | 17 | Cite each included study and present its characteristics. | Table 1 and Supplemental Materials Tables 3 and 4 |
| Risk of bias in studies | 18 | Present assessments of risk of bias for each included study. | Supplemental Materials Tables 1 and 2 |
| Results of individual studies | 19 | For all outcomes, present, for each study: (a) summary statistics for each group (where appropriate) and (b) an effect estimate and its precision (e.g. confidence/credible interval), ideally using structured tables or plots. | Results – Meta-Analysis + Figures 2-5 |
| Results of syntheses | 20a | For each synthesis, briefly summarise the characteristics and risk of bias among contributing studies. | Table 1 |
|  | 20b | Present results of all statistical syntheses conducted. If meta-analysis was done, present for each the summary estimate and its precision (e.g. confidence/credible interval) and measures of statistical heterogeneity. If comparing groups, describe the direction of the effect. | Results – Meta-analysis and Supplemental Materials |
|  | 20c | Present results of all investigations of possible causes of heterogeneity among study results. | Results – Meta-Analysis + Figures 3-5 |
|  | 20d | Present results of all sensitivity analyses conducted to assess the robustness of the synthesized results. | Results – Meta-Analysis and Supplemental Materials - Sensitivity Analysis |
| Reporting biases | 21 | Present assessments of risk of bias due to missing results (arising from reporting biases) for each synthesis assessed. | Supplemental Materials – Funnel Plots & Egger’s tests |
| Certainty of evidence | 22 | Present assessments of certainty (or confidence) in the body of evidence for each outcome assessed. | Not performed |
| **DISCUSSION** | | |  |
| Discussion | 23a | Provide a general interpretation of the results in the context of other evidence. | Discussion |
|  | 23b | Discuss any limitations of the evidence included in the review. | Discussion – Limitations |
|  | 23c | Discuss any limitations of the review processes used. | Discussion – Limitations |
|  | 23d | Discuss implications of the results for practice, policy, and future research. | Discussion |
| **OTHER INFORMATION** | | |  |
| Registration and protocol | 24a | Provide registration information for the review, including register name and registration number, or state that the review was not registered. | Not registered |
|  | 24b | Indicate where the review protocol can be accessed, or state that a protocol was not prepared. | Not publicly available; available upon request |
|  | 24c | Describe and explain any amendments to information provided at registration or in the protocol. | Not applicable |
| Support | 25 | Describe sources of financial or non-financial support for the review, and the role of the funders or sponsors in the review. | Disclosures |
| Competing interests | 26 | Declare any competing interests of review authors. | Disclosures |
| Availability of data, code and other materials | 27 | Report which of the following are publicly available and where they can be found: template data collection forms; data extracted from included studies; data used for all analyses; analytic code; any other materials used in the review. | Data Availability Statement |

**REFERENCES**

1. Barone M, Salzillo R, De Bernardis R, Brunetti B, Tenna S, Persichetti P. Efficacy of Renuvion Helium Plasma to Improve the Appearance of Loose Skin in Patients Undergoing Abdominoplasty After Massive Weight Loss: A Prospective Controlled Randomized Study. *Aesthetic Plast Surg*. Apr 2025;49(8):2260–2268. doi:10.1007/s00266-024-04655-1

2. Ouf MAA, Kishk T, Elnahas MA. Evaluation of Traditional Liposuction, VASER Liposuction, and VASER Liposuction Combined with J-plasma in Management of Gynecomastia. *Plast Reconstr Surg Glob Open*. Nov 2024;12(11):e6277. doi:10.1097/gox.0000000000006277

3. Arshad S, Sin Z, Flores D, Satey S, Mowlavi A. Brazilian buttock lift for the weight-loss patient. *The American Journal of Cosmetic Surgery*. 2023;40(2):132–141.

4. De La Cruz E. Lipoplasty in the overweight patient. *Clinics in Plastic Surgery*. 2024;51(1):29–43.

5. De Souza AST DSA. Brazilian Butt Lift Procedures when the Renuvion APR System was used as an Adjunct Procedure: A Retrospective Chart Review. *J Aesthet Reconstr Surg*. August 11, 2022 2022;8(4:150):1–7. doi:10.36648/2472-1905.8.4.150

6. Doolabh V. A Single-site Postmarket Retrospective Chart Review of Subdermal Coagulation Procedures with Renuvion. *Plast Reconstr Surg Glob Open*. Nov 2019;7(11):e2502. doi:10.1097/gox.0000000000002502

7. Doolabh V, Ruff P. A retrospective chart review of subdermal neck coagulation using helium plasma technology. Review. *Dermatological Reviews*. 2020;1(4):143–150. doi:10.1002/der2.32

8. Driscoll CR, Davidson AL, Marturano MN, Kortesis BG, Bharti G. Expansion of Renuvion Application to Areas Beyond the Submental Region: Review and Experience. Article. *Aesthetic Surgery Journal Open Forum*. 2024;6doi:10.1093/asjof/ojae041

9. Hoyos AE, Perez ME, Cala LC, Dominguez R, Vranis NM, Dayan E. Helium Plasma-Driven Radiofrequency for Skin Contraction: Clinical Use, Safety Recommendations, and Results in High-Definition Body Contouring Surgery. *Aesthet Surg J Open Forum*. 2025;7:ojae118. doi:10.1093/asjof/ojae118

10. Ibrahiem SMS. Aesthetic Nonexcisional Arm Contouring. Article. *Aesthetic surgery journal*. 2022;42(7):NP463–NP473. doi:10.1093/asj/sjac031

11. Khedr MM, Elshawadfy SE. Scarless Enhanced Body Contouring. *Aesthetic Plast Surg*. Jan 2025;49(1):259–268. doi:10.1007/s00266-024-04401-7

12. Kluska M, Deal RC, Summers K, Chang S. A Retrospective Review Comparing Renuvion Helium Plasma Radiofrequency with BodyTite Bipolar Radiofrequency after Liposuction or Body Contouring. *Plastic and Reconstructive Surgery–Global Open*. 2024;12(8):e6024.

13. Lacerna Kimbrell M. Helium Plasma Radiofrequency Brow Lift: Description of a Novel Technique and Retrospective Study on Safety, Efficacy, and Durability. *Aesthetic Surgery Journal Open Forum*. 2025;7:ojaf031. doi:10.1093/asjof/ojaf031

14. Mowlavi A, Talle A, Berri M, Rashid W. Successful Back Contouring With Elimination of Back Rolls Using Ultrasound-Assisted Liposuction and Helium-Activated Radiofrequency. *Aesthet Surg J Open Forum*. Dec 2020;2(4):ojaa036. doi:10.1093/asjof/ojaa036

15. Núñez Villar PJ, Monroy MJ. Lipotransfer Under Ultrasound-guided Helium Pneumodissection With Closed Lipotransfer Equipment. *Plastic and Reconstructive Surgery–Global Open*. 2024;12(12):e6395.

16. Ruff IV PG, Bharti G, Hunstad J, et al. Safety and efficacy of renuvion helium plasma to improve the appearance of loose skin in the neck and submental region. *Aesthetic Surgery Journal*. 2023;43(10):1174–1188.

17. Ruff PG, Doolabh V, Zimmerman EM, Gentile RA. Safety and efficacy of helium plasma for subdermal coagulation. *Dermatological Reviews*. 2020;1(3):108–114.

18. Ruff IV PG, Martinez A, Burpee N. Refining Liposuction Safety and Efficacy: Surgeon Learning-Curve Analysis With Helium-Based Plasma Integration. Oxford University Press US; 2024:ojae063.

19. Ruff P, Parikh R, Mowlavi A, Zimmerman E, Lobowitz J. Safety of Gynecomastia Procedures in Combination with Helium-Based Plasma Technology: A Retrospective Review. *J Aesthet Reconstr Surg*. 2022;8(No.5:152):1–6. doi:10.4172/2472-1905.8.6.152

20. Ruff PG, Vanek P, Nykiel M. Adverse Events of Soft Tissue Coagulation Using a Helium-Based Plasma Technology Alone and in Combination With Ultrasound-Assisted Liposuction. Article. *Aesthetic Surgery Journal Open Forum*. 2022;4doi:10.1093/asjof/ojac064

21. Shridharani SM, Ruff IV PG, Doolabh VB, Zimmerman EM. The safety of contraction of subcutaneous tissue following liposuction procedures. Oxford University Press US; 2024:ojad112.

22. Shridharani SM, Kennedy ML. Safety of Helium-based Plasma Technology for Coagulation of Soft Tissue: A Retrospective Review. Article. *Aesthetic Surgery Journal Open Forum*. 2022;4doi:10.1093/asjof/ojac081

23. Skenderian S, Sin Z, Mirzania H, Mowlavi A. Modified Abdominal Skin Resection: A Novel Approach to High-Definition Body Contouring of the Abdomen. *The American Journal of Cosmetic Surgery*. 2023;40(3):194–203.

24. Sterodimas A, Moutafis A, Nicaretta B, Champsas G. A Prospective Study on Helium-Based Plasma Radiofrequency for Minimally Invasive Breast Lift Scarless Mastopexy. *Aesthet Surg J Open Forum*. 2025;7:ojaf004. doi:10.1093/asjof/ojaf004

25. Sterodimas A, Nicaretta B, Koytsouveli A, Moutafis A, Champsas G. A Prospective Study on Helium-based Plasma Radiofrequency for Management of the Lower Eyelids in Greece. *Plast Reconstr Surg Glob Open*. May 2025;13(5):e6796. doi:10.1097/gox.0000000000006796

26. Sterodimas A, Nicaretta B, Koytsouveli A, Moutafis A, Champsas G. Helium-Based Plasma Radio Frequency Technology for Subdermal Coagulation in the Forehead: A Preliminary, Single-Center Retrospective Study. *Aesthetic Plast Surg*. Mar 21 2025;doi:10.1007/s00266-025-04737-8

27. Tambasco D, Albanese R, Scarabosio A, Tomaselli F, Parodi PC. A three-dimensional approach to male chest enhancement: a surgical algorithm based on 300 cases. *Aesthetic plastic surgery*. 2024;48(18):3646–3655.

28. Tambasco D, Albanese R, Scarabosio A, et al. Ultrasound and Helium Plasma-Assisted Liposuction for Body Contouring: A Single-Retrospective Cohort Study of 639 Patients. *Aesthetic Plast Surg*. Jan 2025;49(1):277–286. doi:10.1007/s00266-024-04367-6

29. Tambasco D, Albanese R, Tomaselli F, Pinto V, Pinelli M, De Santis G. MINIMIZING THIGHPLASTY COMPLICATIONS: A COMBINED APPROACH OF J MEDIAL PATTERN AND HELIUM-PLASMA ASSISTED LIPOSCULPTURE. *Plastic and Reconstructive Surgery*. 2025:10.1097. doi:DOI: 10.1097/PRS.0000000000011962

30. Tambasco D, Tomaselli F, Albanese R. The use of helium plasma cutting device in lipoabdominoplasty: preliminary results. *Aesthetic plastic surgery*. 2024:1–2.

31. Troell RJ, Javaheri S. Combining Third-Generation Ultrasound Liposuction With Helium-Based Plasma Technology Skin Tightening in the Face and Neck. *The American Journal of Cosmetic Surgery*. 2025:07488068251330030. doi:10.1177/07488068251330030

32. Vanek P. A Retrospective Evaluation of the Safety of Combining Helium-Based Plasma Radiofrequency Technology With Ultrasound-Assisted Lipoabdominoplasty. *Aesthet Surg J Open Forum*. 2025;7:ojae116. doi:10.1093/asjof/ojae116

33. Zorrilla AM, Sanchez-Covarrubias AP, Martin K, Rodriguez M. Pain Management and Opioid Consumption After Outpatient Cosmetic Surgery. *The American Journal of Cosmetic Surgery*. 2023;40(3):170–176.

34. Mowlavi A, Farrell J, Talle A, Berri M, Hamlet G. Ultrasound-Assisted Liposuction and Helium-Activated Radiofrequency Skin Tightening for Treatment of Paradoxical Adipose Hyperplasia After Cryolipolysis. *The American Journal of Cosmetic Surgery*. 2020;37(4):168–173.
